# An endovascular porcine model of abdominal aortic aneurysm for interventional radiology research

**DOI:** 10.1186/s41747-025-00673-z

**Published:** 2026-01-26

**Authors:** Marie-Luise Helene Hildegard Ranner-Hafferl, Dilyana Branimirova Mangarova, Jennifer Mein, Jennifer Lilly Heyl, Jana Möckel, Dirk Schnapauff, Timo Alexander Auer, Federico Collettini, Jan Ole Kaufmann, Lisa Christine Adams, Marcus Richard Makowski, Bernd Hamm, Avan Kader, Julia Brangsch

**Affiliations:** 1 Department of Radiology, Charité–Universitätsmedizin Berlin, corporate member of Freie Universität Berlin, Humboldt-Universität zu Berlin, Berlin, Germany; 2 Department of Veterinary Medicine, Institute of Animal Welfare, Animal Behavior and Laboratory Animal Science, Freie Universität Berlin, Berlin, Germany; 3 Berlin Institute of Health (BIH), Berlin, Germany; 4 Department of Diagnostic and Interventional Radiology, Technical University of Munich, Munich, Germany

**Keywords:** Aortic aneurysm (abdominal), Disease models (animal), Endovascular procedures, Extracellular matrix, Swine

## Abstract

**Objective:**

Abdominal aortic aneurysm (AAA) remains a life-threatening condition with few large-animal disease models. We aimed to develop a fully endovascular porcine AAA model for radiology research, reducing surgical trauma and improving reproducibility *versus* laparotomy-based models.

**Materials and methods:**

Fourteen female German Landrace swine (*n* = 14, 30–40 kg) underwent angiography-guided intervention. The animals’ infrarenal aorta was dilated by ~30% via balloon catheter, then collagenase (6,000 IU), elastase (500 IU), and 25% calcium chloride (0.5 mL) were locally incubated to weaken the vessel wall. Eight animals were included in the study; group 1 (*n* = 4) was euthanized at 2 weeks, and group 2 (*n* = 4) at 4 weeks. Aortic diameter was measured weekly by ultrasound; *ex vivo* histology, immunofluorescence, and western blot assessed remodeling and inflammation.

**Results:**

Progressive aneurysm expansion was observed, with diameters of 1.32 ± 0.08 cm (mean ± standard deviation) at 1 week post-intervention, 1.59 ± 0.06 cm at 2 weeks, 1.81 ± 0.10 cm at 3 weeks, and 1.94 ± 0.19 cm at 4 weeks (baseline: 0.74 ± 0.08 cm; *p* < 0.001). Experimental groups’ macrophages increased (group 1, 15.12 ± 3.88%; group 2, 16.65 ± 5.27%) compared to control (0.66 ± 0.27%, *p* = 0.012 and *p* = 0.021, respectively). Vascular smooth muscle cells were reduced across interventional groups (45.97 ± 17.26% *versus* control 80.94 ± 14.26%, *p* = 0.005).

**Conclusions:**

This porcine AAA model replicates human disease features with a fully endovascular workflow, offering a valuable platform for evaluation of novel imaging techniques and interventional therapies.

**Relevance statement:**

This study presents a fully endovascular porcine model of abdominal aortic aneurysm for translational research in interventional radiology and imaging. By enabling aneurysm induction entirely through catheter-based techniques, the model could provide a clinically relevant platform for future evaluation of novel endovascular devices and intraluminal therapeutics.

**Key Points:**

- This study established a fully endovascular, translational porcine model of abdominal aortic aneurysm.
- The model exhibited a significant mean aneurysmal dilation of about 161% at 4 weeks and 107% at 2 weeks.
- Serial ultrasound confirmed consistent aneurysm expansion and reproducible growth patterns in surviving animals.
- *Ex vivo* analyses demonstrated inflammation and extracellular-matrix damage, mirroring key features of human abdominal aortic aneurysm pathology.
- This fully catheter-based workflow provides a practical preclinical platform for evaluating imaging techniques and endovascular therapies.

**Graphical Abstract:**

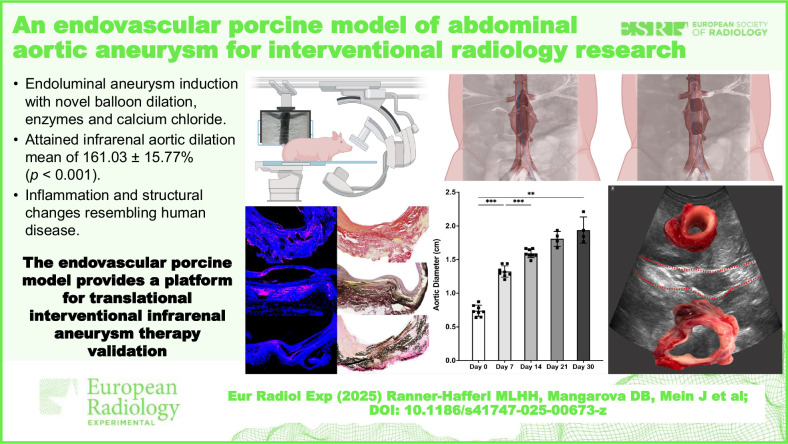

## Background

Abdominal aortic aneurysms (AAA) represent a critical medical concern, defined by an atypical expansion of the aorta. While generally asymptomatic, progressive aneurysmal ectasia is associated with the devastating consequence of aortic rupture, which carries an 80% mortality rate [1, 2]. Despite advancements in surgical interventions becoming more refined and less invasive in the last years, there remains a critical necessity to explore mechanisms that contribute to aneurysm formation and to shift the focus of treatment from surgical to less invasive therapies [2–4]. Animal models are essential for elucidating disease mechanisms and evaluating therapeutic options [5, 6]. While small animal models, notably murine models, are preferred for their cost-effectiveness and genetic tractability, effective large-animal models remain scarce, despite swine models providing significant anatomical and physiological similarity to humans, which is crucial for translational research [7].

In recent years, a handful of porcine AAA models have been developed. Models utilizing elastase perfusion have yielded essential findings regarding histopathological modifications of elastic fibers and alterations in vascular smooth muscle cells (VSMCs) [8]. By combining elastase and collagenase perfusion with mechanical stretching, significant aortic expansion and accompanying extracellular-matrix (ECM) deterioration, notably endothelial dysfunction, neutrophil infiltration, and elastin degradation, can be achieved [9]. However, these techniques require a laparotomy, which causes significant animal and procedural complications. Minimally invasive models, ideal for preclinical testing of novel therapeutic approaches such as 1,2,3,4,6-pentagalloyl glucose-coated balloon catheterization, have yet to be reliably established [10–12].

Building on insights from previous porcine AAA studies by other research groups and our own preliminary experiments, the objective of this study was to establish and validate a purely endovascular, translational porcine model of infrarenal AAA. This design may enable aneurysm formation through standard angiographic access routes, eliminating the need for laparotomy and thereby minimizing procedural trauma, peri-operative complications, and recovery time. The aim in conceiving the model was to provide a reproducible and ethically refined platform for translational preclinical radiology research.

## Materials and methods

### Animals

All *in vivo* procedures of this study were conducted in strict accordance with ARRIVE guidelines and the regulatory standards established by the FELASA [13]. The State Office for Health and Social Affairs of Berlin approved the animal experiments under registration number G 0077/21.

**Fig. 1:**
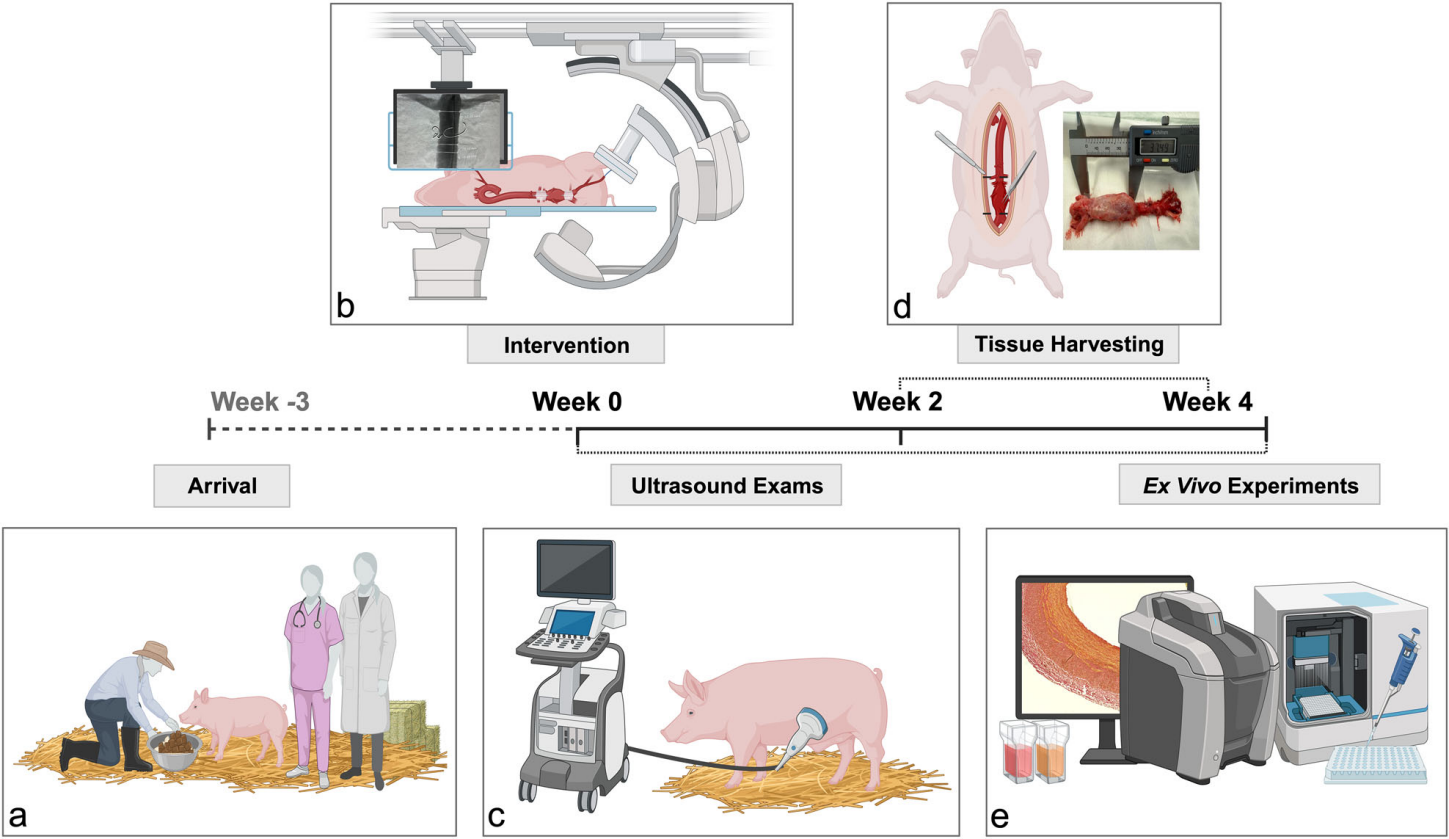
Study design. Schematic overview of the experimental workflow. **a** Animals arrived at the facility 3 weeks prior to intervention for acclimatization. **b** Minimally invasive aneurysm induction was performed under image guidance. **c** Serial ultrasound exams were conducted once weekly for aneurysm monitoring. **d** Animals were randomly allocated to follow-up cohorts at 2 or 4 weeks post-intervention. At the designated time points, euthanasia was performed, and aortic tissue was harvested. **e** *Ex vivo* analyses included histology, immunofluorescence, and western blotting. Created in BioRender. Brangsch, J. (2025) https://BioRender.com/ltxkpf2

This study (Fig. 1) involved fourteen female German Landrace swine (*n* = 14), each weighing between 30 and 40 kg at the time of intervention. Eight animals (*n* = 8) completed the full study protocol and were randomly assigned to two experimental cohorts, with final imaging evaluations conducted at 2 weeks (2 W) and 4 weeks (4 W) post-intervention.

For control comparisons in histopathology and molecular analyses, healthy suprarenal aortic tissue from the same animals was collected and processed. Experienced interventional radiologists performed all interventional procedures.

### Endovascular interventional protocol

Anesthesia was initiated by simultaneous intramuscular administration of atropine (0.05 mg/kg) and azaperone (2 mg/kg), followed by the administration of ketamine (15 mg/kg) and xylazine (2.65–4.3 mg/kg). Propofol (1–3.5 mg/kg) and a fentanyl bolus (10 µg/kg) were administered intravenously (i.v.) before endotracheal intubation and mechanical ventilation setup. Anesthesia was maintained with approximately 1.2% isoflurane and midazolam (2.5–3.5 mg/kg).

A Foley-type urinary catheter was placed to facilitate fluid balance monitoring. A tongue-mounted pulse oximeter was used throughout the procedure to assess pulse rate and oxygen saturation continuously. In addition, an invasive arterial blood pressure monitoring system via the carotid artery was employed for real-time hemodynamic surveillance. Body temperature and electrocardiogram readings were systematically monitored. The mechanical ventilation system enabled continuous tracking of tidal volume and respiratory parameters.

To maintain fluid and analgesic support, continuous Sterofundin® (B. Braun Melsungen AG) and fentanyl infusions (1–10 µg/kg/h) with a minimum flow rate of 5 mL/h were administered, i.v. fentanyl was diluted with saline as required. To maintain the blood pressure of the animals within a consistently physiological range of 100/60–110/75 mmHg, noradrenaline was applied i.v. as a bolus (0.1–1 µg/kg), or alternatively as a continuous infusion (0.01–0.1 µg/kg/h) if mean arterial pressure dropped below 70 mmHg. Additionally, tromethamine (0.2–0.25 mg/kg) and 5% glucose were delivered i.v. to ensure metabolic equilibrium and prevent acidosis. Throughout the procedure, activated clotting time tests, blood gas analyses, and pH measurements were performed at intervals of 30 min or less, along with assessments before and after each interventional procedure and following the administration of heparin.

**Fig. 2:**
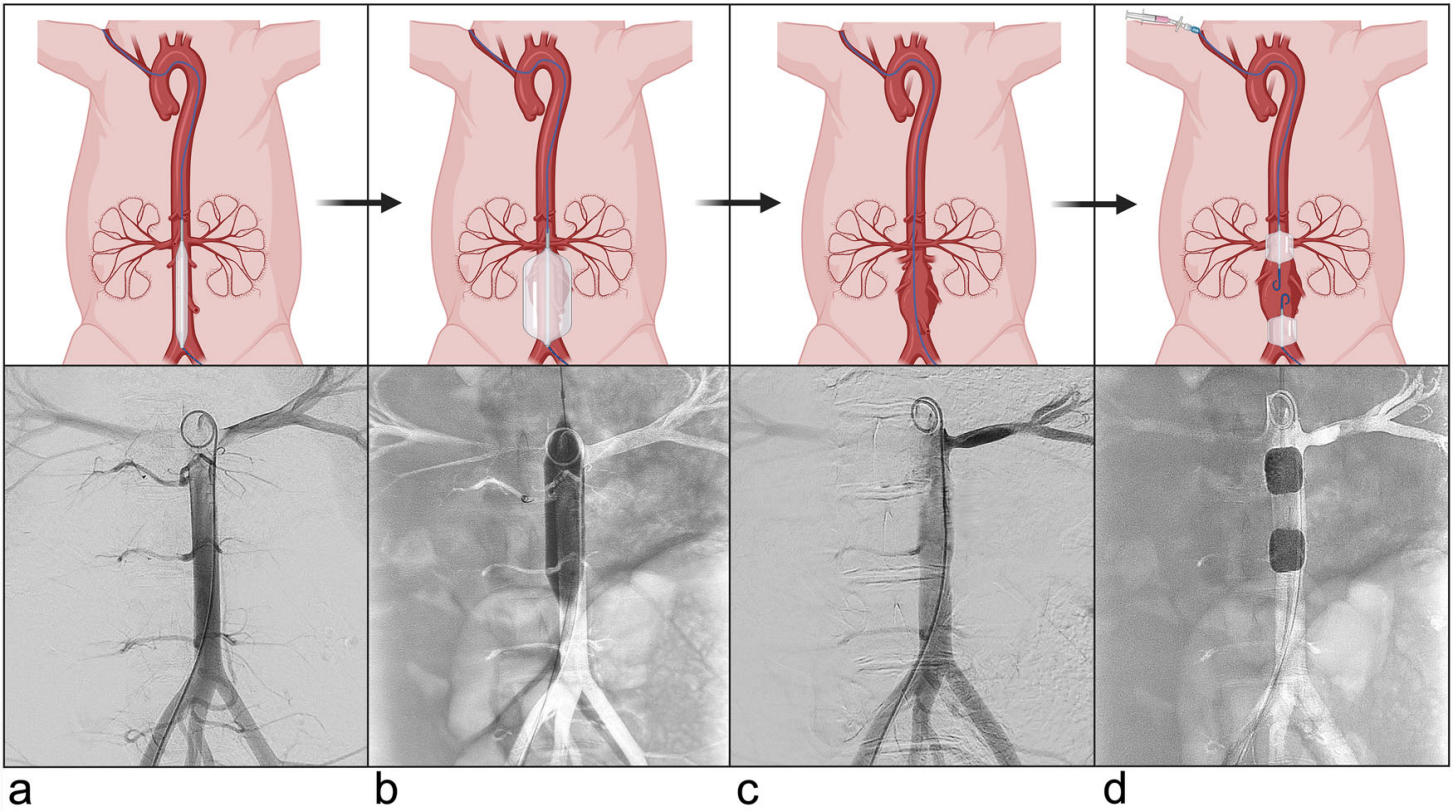
Endovascular protocol for abdominal aortic aneurysm induction. Schematic (top) and angiographic (bottom) images showing: (**a**) baseline aortography, (**b**) balloon dilation (14 mm, 10 min, 6–8 atm), (**c**) post dilation aortography to confirm successful stretching, (**d**) enzymatic incubation with collagenase and elastase (6,000 IU and 500 IU, respectively; 20 min)/calcium chloride application (25%, 0.5 mL, 15 min) under cranial/caudal balloon catheter facilitated isolation of infrarenal aorta segment. Created in BioRender. Brangsch, J. (2025) https://BioRender.com/ltxkpf2

Before instrumentation, each animal was shaved, aseptically cleansed, and draped under sterile conditions. Following ultrasound-guided vascular puncture, a 6 F angiographic sheath (Radifocus^TM^, Terumo Medical Corp.) was introduced into the right common carotid artery, and an 8 F sheath (Radifocus^TM^, Terumo Medical Corp.) was placed in the right common femoral artery. Angiographic visualization of the abdominal aorta between the renal arteries and the aortic bifurcation was achieved (Fig. 2a) through the utilization of 4 F pigtail catheters (TEMPO^TM^, Cordis Corp.) and iomeprol (Bracco Imaging S.p.A.; ~600 mg I/kg) as a contrast agent, and the baseline diameter was measured. An initial dose of 5,000 IU of heparin was administered through the femoral sheath.

In the initial step for AAA induction, mechanical dilation of the infrarenal abdominal aorta was performed (Fig. 2b). Using a 14 × 40 mm balloon catheter (Atlas^TM^, Becton Dickinson GmbH), the aorta was dilated for 10 min at a pressure of 6–8 atm, aiming for a dilation of 30% compared to baseline measurements. After 10 min, the balloon catheter was deflated and subsequently removed. Aortography was performed again, and the aortic diameter was measured to confirm a successful dilation (Fig. 2c).

Thereafter, 5.5‒6 F Arterial Embolectomy Catheters (Fogarty®, Edwards Lifesciences Corp.) were strategically positioned between the first two pairs of lumbar arteries caudal to the renal artery and consequently inflated (Fig. 2d). The efficacy of the induced occlusions was fluoroscopically determined by iomeprol administered via a 2 F Microcatheter (Progreat^TM^, Terumo Medical Corp.) which was inserted through the lumen of the caudal Fogarty® balloon. Subsequently, 6,000 IU of collagenase (Type 1, CLS 1) and 500 IU of elastase (10 mg ≥ 200 U/mg, lyophilized) dissolved in 1 M Tris-HCl buffer were administered through the microcatheter and incubated for 20 min. The enzymes were then carefully removed via a 60 mL saline flushing, and the balloons were deflated for reperfusion of the aorta for 10 min. Thereafter, the Fogarty® Catheters were inflated once more and positioned as described above. Then, 0.5 mL of a 25% calcium chloride (CaCl_2_) solution, dissolved in sterile *aqua ad injectabilia* was administered, incubated for 15 min, and then removed via saline flushing.

The puncture sites were occluded using a vascular closure device (Angio-Seal®, Terumo Medical Corp.) and additional skin sutures. Following the procedure, animals were injected with i.v. buprenorphine (0.03 mL/kg) and received fentanyl (50 µg/h) through transdermal patches for 6 days. To mitigate the risk of postoperative infections and alleviate discomfort, the animals were provided a treatment regimen comprising a sulbactam/ampicillin (0.05/0.025 mg/kg) and a metronidazole (15 mg/kg) i.v. infusion in conjunction with an intramuscular metamizole (25–30 mg/kg) injection. Metamizole application was continued orally twice daily for 1 week following the intervention. Over the next week, the animals underwent twice-daily examinations, including heart auscultation, blood pressure measurement, wound inspection, temperature monitoring, and respiratory rate assessment. These evaluations were then conducted once daily during the following weeks. Additionally, nightly checks on temperature and activity were performed every 3 h starting at 11 p.m. from the time the swine underwent interventions until euthanasia, utilizing thermal imaging, night vision cameras, and ear tag BioCV® Livestock Monitoring (BioCV® GmbH).

### Ultrasound imaging

Ultrasound examinations were conducted before the intervention and then weekly to monitor AAA growth and evaluate rupture risk. The probe was placed on the right flank, approximately 5 cm cranial to the anterior superior iliac spine. After locating the right kidney to standardize imaging planes, both the abdominal aorta and the inferior vena cava were visualized. For each time point, three images per animal were analyzed using Horos software (Version 4.0.0; The Horos Project). The aorta was identified and measured in each image, and the values were averaged to obtain a representative diameter.

### Harvest procedure

The eight animals (*n* = 8) that reached the estimated endpoints of the study were euthanized either at 2 W (*n* = 4) or 4 W (*n* = 4) after the interventional procedure. Euthanasia was conducted under deep i.v. propofol anesthesia (20 mg/kg/h) via i.v. administration of pancuronium (0.05–0.07 mg/kg) followed by potassium chloride (149–199 mg/kg) i.v. injection.

Infrarenal aortic segments as well as suprarenal internal control tissue were excised during necropsy for histopathology, immunofluorescence (IF) and western blotting (WB) analyses, caliper measurements of the external aortic diameter were performed. Frozen and formaldehyde-fixed samples were embedded before slicing into 10 µm sections and mounted onto adhesive slides for additional analysis. Specimens designated for WB were stored at -20 °C without any additives.

### Histopathology

Paraffin-embedded tissue sections underwent deparaffinization and rehydration before histopathological processing. Elastica-Van Gieson staining was utilized to demonstrate elastin, whereas Picro-Sirius Red staining was employed to delineate collagenous structures. Von Kossa staining was performed to evaluate calcium mineralization.

Microscopic examination was performed at a magnification of × 10 utilizing the Keyence BZ-X800 Series microscope (Keyence Corp.). The Keyence Hybrid Cell Count software (Version 2.1.1; Keyence Corp.) facilitated the quantification of the calcium proportion relative to the total sample area, thereby enabling the computation of the calcified-to-non-calcified tissue ratio. To assess elastic fiber and collagen degradation, fragmentation of elastic fibers/collagen was evaluated similarly.

Morphometric analyses were performed using ImageJ software (1.54p, Java SE 23; National Institutes of Health).

### Immunofluorescence

Frozen tissue sections were fixed using acetone, followed by a washing protocol in phosphate-buffered saline (PBS). The sections were incubated with primary antibodies overnight, secondary antibodies, Goat anti-Mouse IgG (H + L) or Donkey anti-Rabbit IgG (H + L) (Highly Cross-Adsorbed Secondary Antibody, Alexa Fluor™ 647, #A-21236, #A-31573, 1:1000; Invitrogen, Thermo Fisher Scientific Inc.) were used for binding detection. Mounting was done with 4′,6‑diamidino‑2‑phenylindole‒DAPI staining solution.

Imaging of the entire aorta was conducted utilizing a Keyence BZ-X800 fluoroscope (Keyence Corp., Osaka, Japan), with images captured at × 10 magnification and stitched together to provide comprehensive visualization. Macrophage content was evaluated using Galectin-3 (Gal-3) antibody (#14979-1-AP, 1:100; Proteintech Group Inc.). Assessment of myofibroblast formation was carried out utilizing an α-smooth-muscle-actin (α-SMA) antibody (#sc-53142, 1:100; Santa Cruz Biotechnology Inc.).

Quantitative analysis was performed using the Keyence Hybrid Cell Count software (Version 2.1.1; Keyence Corp.) with automatic thresholding for hue, brightness, and color tolerance to maintain consistent segmentation across samples.

Gal-3 and α-SMA signals were expressed as area fractions of positive staining relative to the total DAPI-positive area within the obtained image.

### Western blotting

Frozen biological specimens obtained from both infrarenal and suprarenal segments of the aorta were rinsed in PBS and then lysed in a buffer formulation before homogenization and subsequent incubation at 4 °C for 90 min.

Following the incubation period, the samples underwent centrifugation and were then diluted to a final concentration of 1.25 mg/mL. As analysis was performed using the Jess^TM^ Simple Western automated capillary-based size separation and nano-immunoassay system, the lysates were denatured before loading into 12–230 kDa Jess^TM^ separation module plates.

Chemiluminescent detection was performed using Jess^TM^ Anti-Rabbit Detection Modules for primary antibody Gal-3 (#14979-1-AP, 1:10; Proteintech Group Inc.). Jess^TM^ Anti-Mouse Detection Modules were employed for quantification of α-SMA (#sc-53142, 1:1000; Santa Cruz Biotechnology Inc., Dallas, TX, USA).

Signal intensity from each sample and the digital blot images were collected using Compass for Simple Western software (Version 7.0.0; ProteinSimple^TM^ Biotechne Corp.).

For both WB and IF experiments, antibody cross-reactivity with porcine antigens was verified based on manufacturer data and preliminary test staining. Each assay included positive controls consisting of aortic tissue sections with verified expression of the target protein, and negative controls in which either the primary or secondary antibody was omitted to confirm signal specificity.

### Statistical analyses

Statistical evaluations were performed using IBM SPSS Statistics Software (Version 29.0.0, IBM Corp.). Data visualization was facilitated using GraphPad Prism (Version 10.4.1, Dotmatics), while workflow illustrations and study design schematics were created using BioRender (Version 2025; Science Suite Inc.). Graphs are shown as bar charts with error bars, with individual data points overlaid as dots, squares, and triangles.

Data are reported as mean ± standard deviation. Statistical significance was defined at α < 0.05; exact *p*-values and 95% confidence intervals are provided for all primary outcomes.

For ultrasound-derived diameter measurements, a two-way ANOVA (time × group) was employed, followed by Tukey’s post hoc correction. Prior to analysis, normality was confirmed using the Shapiro–Wilk, D’Agostino–Pearson, Anderson–Darling and Kolmogorov–Smirnov tests, and homoscedasticity was verified via Spearman’s rank correlation. Results were confirmed by a linear mixed-effects model (time as fixed effect, animal as random effect).

For all other continuous endpoints, where heterogeneity of variances was anticipated, a Welch or Brown–Forsythe ANOVA was used, followed by Dunnett’s T3 multiple-comparison tests. For these analyses, normality (Shapiro–Wilk) and variance homogeneity (Brown–Forsythe test or Spearman correlation) were likewise assessed.

A per-animal dataset of quantitative outcome measures used for statistical analysis is provided in the Supplementary material (Table S10).

## Results

Overall, 14 animals underwent AAA induction using the novel minimally invasive approach. In eight animals (~57%), successful aneurysm formation was achieved.

Five animals (~36%) were euthanized prematurely due to procedure-related complications, and one animal did not develop an AAA due to material failure during mechanical dilation but was euthanized at the study endpoint at 4 weeks.

Two animals had to be euthanized due to aortic rupture, either during the intervention (*n* = 1) or 24 h after the intervention (*n* = 1), while three animals were euthanized following the onset of hind-limb paresis induced by complications from balloon catheter malfunction, leading to CaCl_2_/enzyme flushing of the animal’s vascular system.

### Aortic diameter measurements and comparative analysis

During the intervention for AAA induction, the aortic diameter was mechanically dilated by 0.37 ± 0.38 cm, achieving a 27.05 ± 3.27% (*p* = 0.013) expansion of the segment between the renal arteries and the inferior mesenteric artery. This initial mechanical dilation served as the foundation for subsequent time-dependent expansion following enzyme and CaCl_2_ application. Measurements were standardized to ensure consistent dilation across animals.

Following AAA induction, aortic diameter was assessed weekly using ultrasound and subsequently confirmed post-mortem through measurements of paraffin-embedded tissue sections and gross caliper measurements performed *in situ* during necropsy. The baseline aortic diameter determined by ultrasound before intervention was 0.74 ± 0.08 cm.

**Fig. 3:**
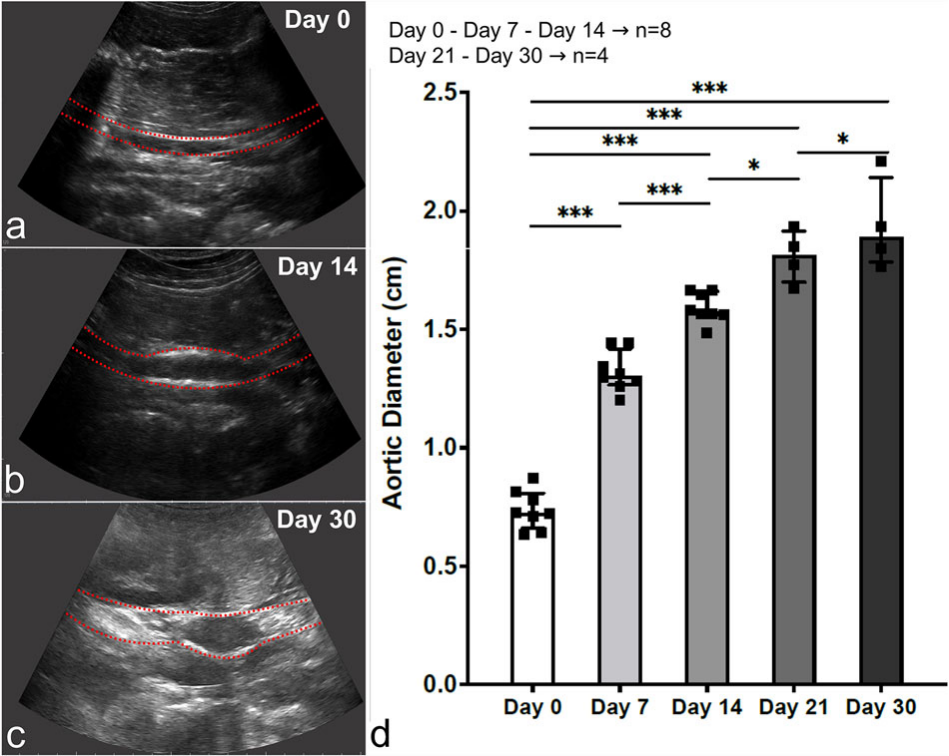
Longitudinal ultrasound assessment of aortic diameter post-abdominal aortic aneurysm induction. **a**–**c** Representative B-mode ultrasound images of the infrarenal aorta at day 0 (**a**), day 14 (**b**), and day 30 (**c**). Red dotted lines indicate aortic wall boundaries. **d** Quantification of aortic diameter over time showing progressive expansion: 1.32 ± 0.08 cm on day 7, 1.59 ± 0.06 cm on day 14, 1.81 ± 0.11 cm on day 21, and 1.94 ± 0.19 cm on day 30, compared to baseline (0.74 ± 0.08 cm; *p* < 0.001 *versus* day 7, 14, 21 and 30). Individual data points represent single animals (squares, *n* = 8): *n* = 8 at day 0, day 7 and day 14; *n* = 4 at day 21 and day 30. Statistical significance: *** *p* < 0.001, * *p* < 0.05

Ultrasound examinations were repeated weekly, revealing a statistically significant progressive increase in aortic diameter. On day 7, the diameter reached 1.32 ± 0.08 cm (+79.23 ± 4.80% *versus* day 0; *p* < 0.001). By day 14, it further increased to 1.59 ± 0.06 cm (+107.14 ± 4.04% *versus* day 0; *p* < 0.001). A diameter of 1.81 ± 0.11 cm was measured on day 21 (+142.46 ± 8.66% *versus* day 0, *p* < 0.001). At day 30, measurements showed 1.94 ± 0.19 cm (+161.03 ± 15.77% *versus* day 0, *p* < 0.001) (Fig. 3).

**Fig. 4:**
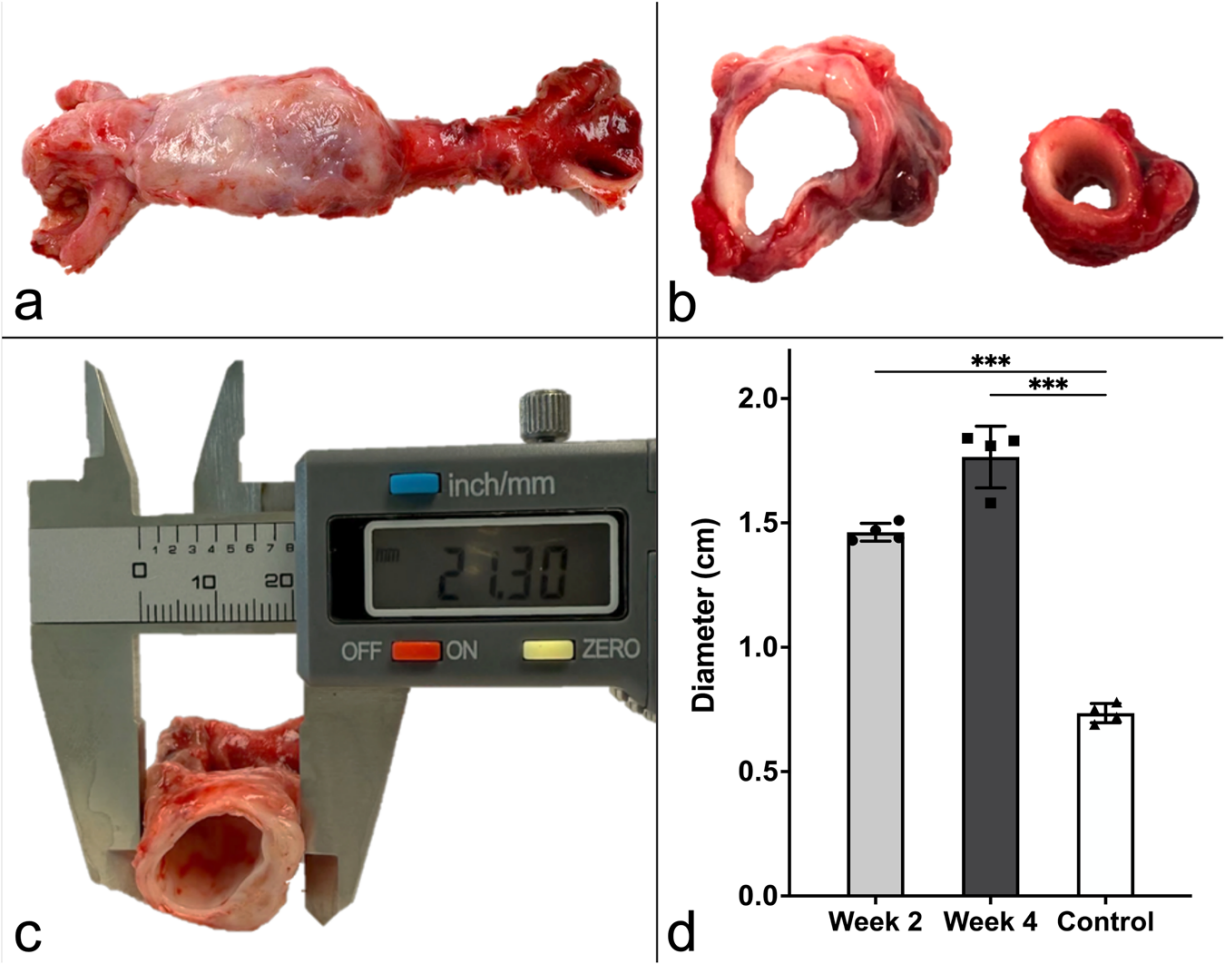
Aortic diameter measurements following endovascular abdominal aortic aneurysm induction. **a**, **c** Representative images and caliper measurement of aneurysmal aortic segments 4 weeks (4 W) post-intervention. **b** Transverse sections illustrate dilated *versus* control suprarenal aortae. **d** Measured *ex vivo*, 2 weeks (2 W) post-intervention, diameter reached 1.46 ± 0.04 cm (97.30 ± 5.41% above baseline, *p* < 0.001), and at 4 W, 1.77 ± 0.12 cm (139.19 ± 16.21%, *p* < 0.001). Baseline control: 0.74 ± 0.04 cm. Individual values: *n* = 4 for 2 W (dots); *n* = 4 for 4 W (squares); *n* = 4 for controls (triangles). Statistical significance: *** *p* < 0.001. 2 W, Two weeks; 4 W, Four weeks

Post-mortem analyses confirmed these findings. At 2 W post-intervention, the mean aortic diameter was 1.46 ± 0.04 cm, corresponding to a 97.30 ± 5.41% increase over control (*p* < 0.001). In animals euthanized at 4 W, the diameter reached 1.77 ± 0.12 cm, representing a 139.19 ± 16.21% increase compared to baseline (*p* < 0.001) (Fig. 4).

### Histopathological analysis of aortic wall composition

**Fig. 5:**
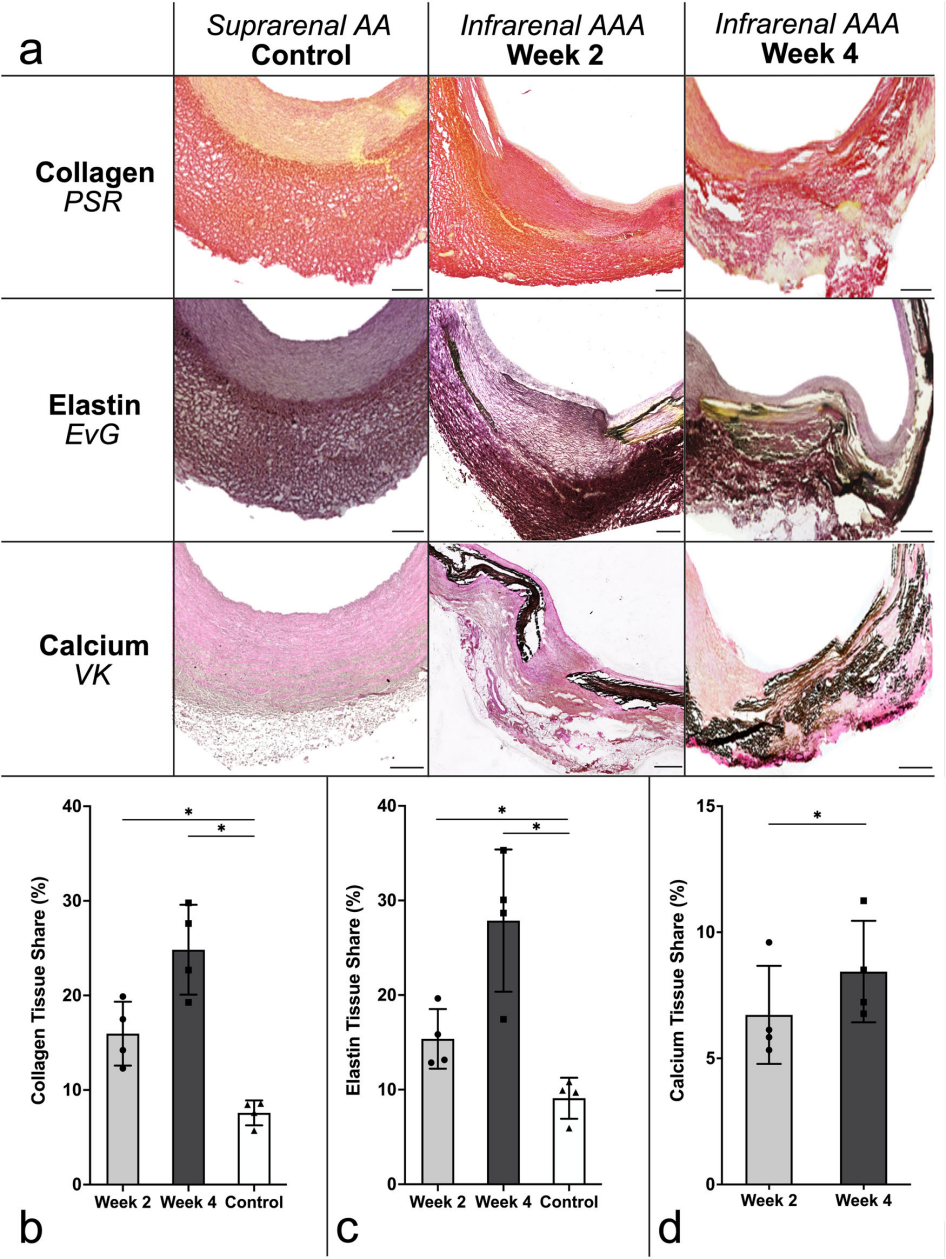
Histological assessment of aortic wall remodeling. **a** Representative histological sections of abdominal aortic tissue stained with Picro-Sirius Red (top row, PSR), Elastica-Van Gieson (middle row, EvG), and Von Kossa (bottom row, VK), comparing suprarenal control (left), 2 weeks (2 W, middle), and 4 weeks (4 W, right) post-intervention. Scale bars: 500 µm. Quantification of (**b**) collagen content (2 W: 15.97 ± 3.38%; 4 W: 24.84 ± 4.75%; control: 7.58 ± 1.32%; *p* = 0.025, and *p* = 0.014, respectively), (**c**) elastic fiber fragmentation (2 W: 15.37 ± 3.15%; 4 W: 27.87 ± 7.52%; control: 9.1 ± 2.17%; *p* = 0.049, and *p* = 0.040, respectively), and (**d**) calcium deposition (2 W: 6.73 ± 1.94%; 4 W: 8.44 ± 2.01%; *p* = 0.041). Quantified with Keyence Hybrid Cell Count (autothreshold; Version 2.1.1, Keyence Corp.). Individual values: *n* = 4 for 2 W (dots); *n* = 4 for 4 W (squares); *n* = 4 for controls (triangles). Statistical significance: * *p* < 0.05. 2 W, Two weeks; 4 W, Four weeks

Histopathological evaluations focused on the assessment of calcium deposition, aortic wall integrity evaluation, and the examination of collagen and elastic fiber remodeling (Fig. 5). Employing hybrid cell counting, calcium deposition was quantified, revealing a prevalence of 8.44 ± 2.01% for the group observed at 4 W, and 6.73 ± 1.94% (*p* = 0.041; 2 W *versus* 4 W) for the cohort evaluated at 2 W, within the infrarenal abdominal aortic wall, confirming progressive calcification. In contrast, the suprarenal regions showed an evident absence of detectable calcium deposits.

The infrarenal aorta exhibited significant deterioration of both collagen and elastin, alongside the marked increase in aortic diameter. 4 W post-intervention an elastin fragmentation of 27.87 ± 7.52% (*p* = 0.04) and collagen reorganization severity of 24.84 ± 4.75% (*p* = 0.014) were recorded, suggesting extensive structural remodeling within the aortic wall when compared to control segments (control 9.1 ± 2.17% for elastic fibers, and 7.58 ± 1.32% for collagen). In animals assessed 2 W post-intervention, 15.37 ± 3.15% (*p* = 0.049) of elastic fibers were fragmented, and 15.97 ± 3.38% of the aortic wall’s collagen was fragmented (*p* = 0.025 *versus* control).

### WB and IF analysis of inflammation and vascular remodeling

**Fig. 6:**
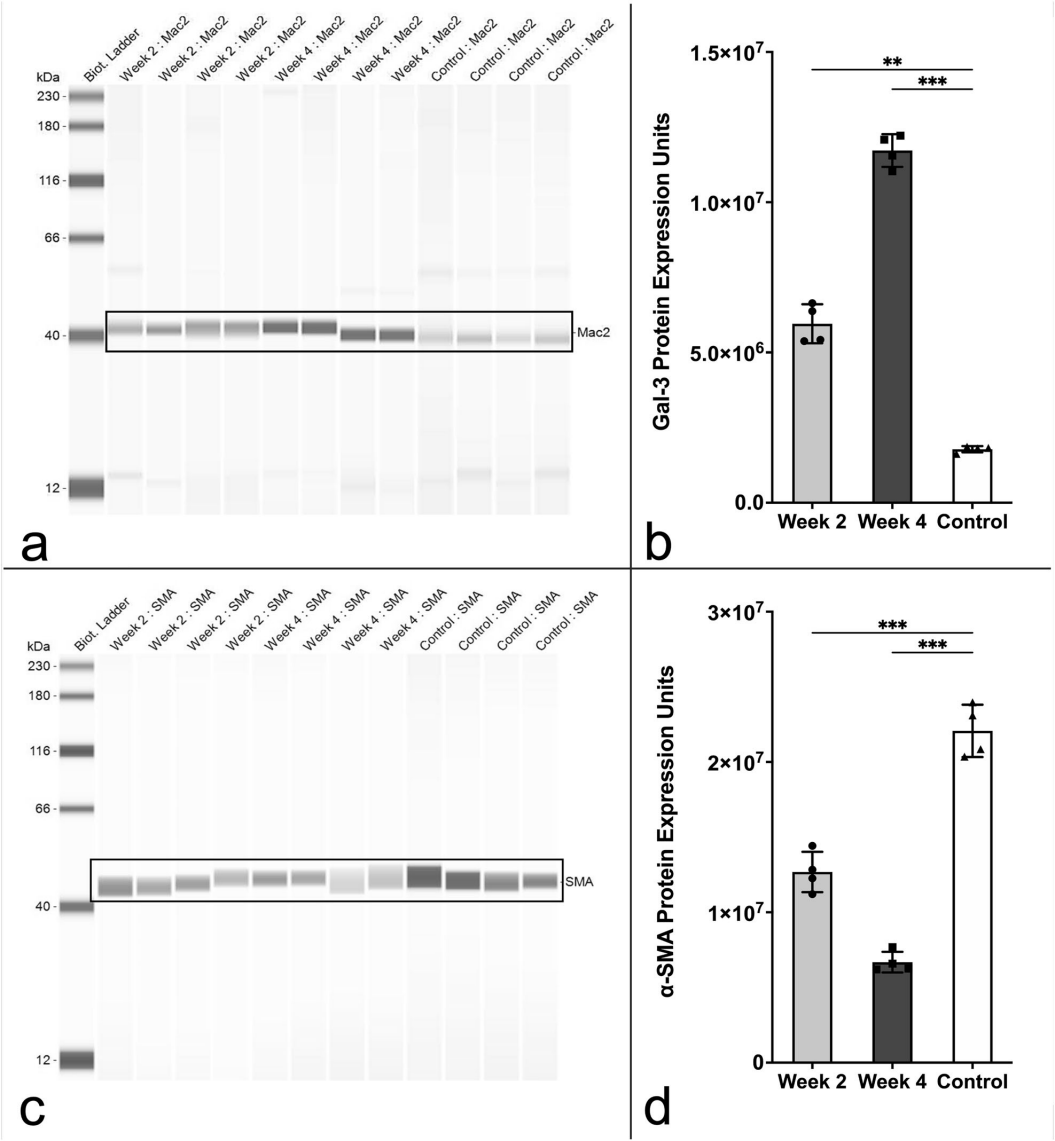
Western blot analysis of inflammatory and structural protein expression. **a** Representative western blot showing Galectin-3/Mac2 (Gal-3) expression in aortic tissue at 2 weeks (2 W) and 4 weeks (4 W) post-intervention *versus* control. **b** Quantification of Gal-3 levels reveals significant upregulation at 2 W (46.55 ± 7.31%) and 4 W (87.22 ± 11.14%) compared to control (18.86 ± 10.37%; *p* < 0.001). **c** WB showing α-smooth-muscle-actin (α-SMA) expression in the same groups. **d** Quantification shows reduced α-SMA expression at 2 W (52.79 ± 14.99%) and 4 W (39.16 ± 17.52%) *versus* control (80.94 ± 14.26%; *p* = 0.005 and *p* < 0.001, respectively). Individual values: *n* = 4 for 2 W (dots); *n* = 4 for 4 W (squares); *n* = 4 for controls (triangles). Statistical significance: ** *p* < 0.01, *** *p* < 0.001. 2 W, Two weeks; 4 W, Four weeks; α-SMA, α-smooth-muscle-actin; Gal-3, Galectin-3

To evaluate macrophage expression, vascular remodeling, immune response, and ECM degradation, WB protein analysis was conducted utilizing antibodies targeting the macrophage marker Gal-3 as well as the VSMC biomarker α-SMA (Fig. 6).

WB analysis quantified α-SMA protein expression of 45.97 ± 17.26% (compared to control 80.94 ± 14.26%, *p* = 0.005, normalized to peak protein expression) for animals subjected to interventional procedures. Comparison of the different time points of analysis (2 W *versus* 4 W) reveals that the group euthanized 2 W after AAA induction demonstrated a higher preservation of α-SMA (2 W: 52.79 ± 14.99% *versus* 39.16 ± 17.52% 4 W, *p* < 0.001).

**Fig. 7:**
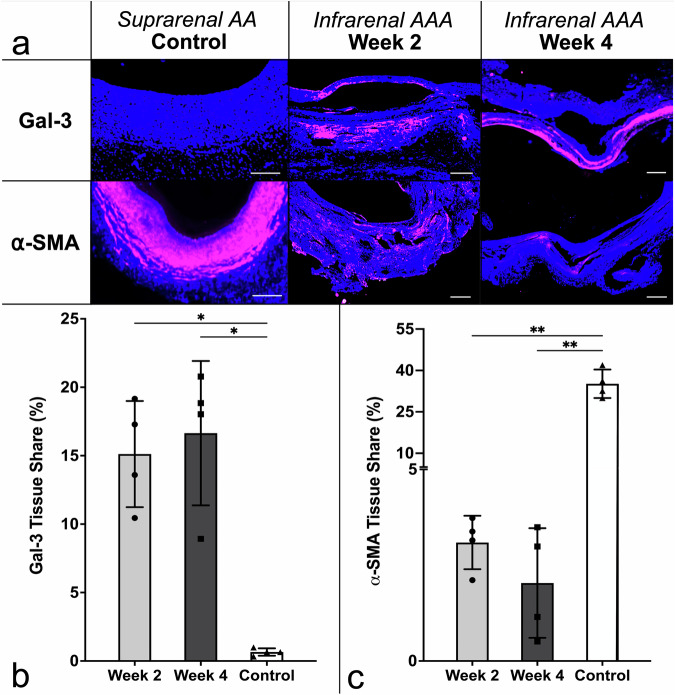
Immunofluorescence analysis of macrophage infiltration and vascular smooth muscle cell loss. **a** Representative images of aortic tissue stained for Galectin-3 (Gal-3, magenta), α-smooth-muscle-actin (α-SMA; magenta), and DAPI (blue), in control, 2 weeks (2 W), and 4 weeks (4 W) post-intervention samples. Scale bars: 500 µm. **b** Gal-3-positive area increased significantly at 2 W (15.12 ± 3.88%) and 4 W (16.65 ± 5.27%) *versus* control (0.66 ± 0.27%; *p* = 0.012, and *p* = 0.021, respectively). **c** α-SMA-positive area decreased at 2 W (3.09 ± 0.70%) and 4 W (2.04 ± 1.43%) *versus* control (35.18 ± 5.15%; *p* = 0.003 for both comparisons). Quantified with Keyence Hybrid Cell Count (autothreshold; Version 2.1.1, Keyence Corp.), values signify positive area fraction. Individual values: *n* = 4 for 2 W (dots); *n* = 4 for 4 W (squares); *n* = 4 for controls (triangles). Statistical significance: * *p* < 0.05, ** *p* < 0.01. 2 W, Two weeks; 4 W, Four weeks; α-SMA, α-smooth-muscle-actin; Gal-3, Galectin-3

Both experimental groups at 2 W and 4 W post-procedure exhibited upregulated Gal-3 expression, as demonstrated by IF (15.88 ± 4.36%) compared to controls (0.66 ± 0.27%, *p* = 0.021; Fig. 7).

## Discussion

This study established a novel minimally invasive methodology for inducing AAAs in a porcine large-animal model, demonstrating consistent aneurysm expansion over time. Ultrasound confirmed progressive dilation, with post-mortem analysis validating the findings. Molecular and structural analyses confirmed that the model reproduces essential pathological features of human AAA, establishing its suitability for translational research applications.

The purely endoluminal, balloon-based mechanical dilation performed via right common femoral access has not, to our knowledge, been previously implemented and confers significant advantages in terms of achievable aneurysm size. The ability to generate larger aneurysms is particularly relevant for interventional device testing, as it allows the use of standard human-scale endovascular instruments and stent graft systems. In swine, the physiological infrarenal aortic diameter typically ranges from 0.6 to 0.8 cm in juvenile animals and up to 1 cm in mature males [14, 15]. When infrarenal aneurysmal expansion remains below 1.5 cm, access and deployment of human-grade endovascular devices become technically challenging or infeasible due to size constraints, limiting translational applicability [16, 17]. Therefore, achieving aneurysm diameters exceeding this threshold increases the model’s translational relevance for preclinical evaluation of endovascular prostheses and local therapeutic delivery systems.

In the model described by De Leo et al [18], AAA induction in swine was accomplished with few (*n* = 3 of *n* = 12) periprocedural losses, but only a modest mean aortic diameter growth of ~27% at 21 days was reported. In contrast, in the present project, expansions exceeding 160% of baseline were achieved without the need for laparotomy. The greater dilation observed is attributed to mechanical stretching performed entirely intraluminally, without the need for surgical exposure of the infrarenal aorta. After procedural refinements, our approach demonstrated a minimally invasive profile comparable to previously described, safely reproducible models, such as that reported by De Leo et al [18], while enabling the formation of substantially larger aneurysms.

Lederman et al [19] were the first to establish a purely endovascular porcine AAA model, using 25% CaCl_2_ or 40.8 IU elastase infused into the infrarenal aorta, achieving diameter increases of up to 100% after 4 W. Their innovative approach marked a significant step toward minimally invasive large-animal aneurysm models. However, limitations such as adverse effects at higher CaCl_2_ doses, elastase variability, small and heterogeneous group sizes, and protocol changes affected reproducibility. In our study, we retained a modified extension of their endovascular access technique but incorporated collagenase as was used in some open surgical models, while adding a novel approach to minimally invasive mechanical dilation. While Lederman et al [19] emphasized biomechanical properties, we focused on histopathological, immunological, and ECM-related changes to improve translational applicability.

Cullen et al [20] advanced the swine AAA model through a synergistic approach that incorporates balloon angioplasty, elastase, and collagenase perfusion, topical application of elastase, and oral administration of β-aminopropionitrile (BAPN). This comprehensive strategy resulted in the development of significant infrarenal aneurysms. Their model effectively replicates crucial characteristics of human AAAs and serves as a robust framework for the investigation of pathogenesis and therapeutic strategies. However, it is essential to note that the model is constrained by inconsistencies in BAPN administration and variations in the quality of elastase perfusion. Consequently, we opted not to utilize BAPN, as we achieved sufficient dilation and immunological similarity to human AAAs through our interventions alone. By avoiding the additional risks and heterogeneity associated with incorporating another oral treatment, we improved the reproducibility of the model by Cullen et al [20].

Compared with previous studies, our model achieved greater aneurysm expansion than previously described open or hybrid techniques. De Leo et al [18] induced AAA in twelve pigs, achieving aneurysm formation in nine (procedural success rate of 75%), though the extent of aortic dilation observed was relatively modest. Conversely, Lederman et al [19] reported pronounced dilation, but the actual procedural success rate is difficult to evaluate, as nine of eighteen pigs were used for protocol fine-tuning and, after two dropouts, among the remaining eight, two served as controls. Cullen et al [20] used 33 swine in total, with twelve undergoing surgeries alone and 21 receiving BAPN orally and being put through surgery. Several animals were excluded or lost during the procedure or early follow-up, and data were ultimately analyzed from only 19/33 pigs. Although substantial dilation was reported in the BAPN-treated group, the high number of dropouts and exclusions makes the true procedural success rate difficult to ascertain.

In this study, eight of 14 animals (57%) successfully completed the protocol and reached the planned endpoints, while six (43%) did not.

Five sequential animal cohorts were evaluated with predefined 2- or 4-week follow-up periods. Procedural complications, including intraoperative rupture and hind-limb paresis, were observed in the early cohorts. In contrast, all animals in the final two cohorts successfully completed both the intervention and follow-up without any adverse events, indicating progressive refinement and stabilization of the experimental protocol. The higher dropout rate observed in the early cohorts primarily reflected the procedural learning curve inherent to the fully catheter-based workflow. After optimization of balloon inflation pressure, standardization of catheter selection, and implementation of a uniform procedural protocol, no further ruptures or neurological complications were observed, underscoring the safety and reproducibility of the refined technique.

The primary advantage of this newly validated model lies in the fact that our methodology, as opposed to those developed by, amongst others, De Leo et al and Cullen et al, can be implemented without the requirement for open surgery [8, 9, 18, 20]. An abdominal incision and the exposure of the infrarenal abdominal aorta result in a significantly larger risk area susceptible to infections and hernias during and after the surgical process. In contrast, our purely interventional model necessitates only two minor arterial incisions, which remain barely discernible and heal rapidly with minimal risk of infection due to the small entry port for pathogens and the limited trauma inflicted on surrounding tissue.

The minimally invasive workflow shortened procedural duration and thereby reduced anesthetic exposure, which poses an important benefit in swine, which tolerate prolonged anesthesia poorly [19]. On veterinary assessment at 24 h post-intervention, animals exhibited no overt signs of pain and required no escalation of analgesia. The same was observed on day 6 when fentanyl patches were removed. These findings are consistent with the minimal tissue trauma inherent to the induction technique [21].

A key challenge in developing a clinically relevant large-animal model for AAA research is balancing the need for accelerated aneurysm formation within the experimental setting with the slow, protracted nature of the disease in humans [22–24]. Elastase is widely used to induce AAAs by fragmenting elastic fibers, a key structural component of the aortic wall. This approach has proven effective in rodent models, particularly in rats and mice, where elastase perfusion significantly influences both the incidence and diameter of AAAs [25–28]. The addition of CaCl_2_ has been shown to enhance these models by promoting vascular mineralization and calcium plaque formation, although CaCl_2_ alone does not substantially affect morphometric AAA magnitude [29, 30].

Histology in this study’s model revealed severe elastic fiber fragmentation and disorganized collagen, confirming active ECM degradation. Calcification was also present, consistent with the medial elastin loss and VSMC apoptosis, which contribute to disease progression by reducing structural support and limiting ECM repair [31–34]. While calcification can locally stiffen the vessel, it did not prevent further expansion when used in moderation [35]. Macrophage upregulation leading to elastin and collagen breakdown, immune cell infiltration driving the release of cytokines, along with proteolytic enzymes, as seen in our model, closely reflects human AAA pathology [36, 37]. These findings validate the model’s relevance for studying disease mechanisms and evaluating therapeutic approaches.

This study has limitations. It was restricted to female animals because bladder catheterization, which was necessary for further studies on these swine (not described here), in males poses significant anatomical difficulties, which would subject the animals to undue strain. Follow-up projects utilizing male swine are planned as warranted by the sex dimorphism of AAA disease. Our study’s use of young animals (12 weeks) does not reflect the typical age of AAA onset in humans, and the small sample size (*n* = 14) reduces statistical robustness.

A high dropout rate due to technical difficulties and biological variability posed an additional challenge. Hind-limb paresis was observed (*n* = 3) after thrombotic embolisms. Balloon positioning and inflation pressure required individual adjustments to accommodate anatomical differences, limiting procedural standardization. Furthermore, leakage or systemic spread of enzymes or CaCl_2_ could cause neuromuscular complications, underscoring the need for precise and controlled application.

Dropouts in the initial project phase reflected procedural errors. Once interventional, anesthetic, and ventilatory parameters were adjusted and standardized, dropout rates fell and remained low, indicating the procedure itself is not inherently high risk.

In conclusion, we presented a novel endovascular porcine model of infrarenal AAA induction using balloon-based mechanical dilation. *Ex vivo* analyses recapitulated key features of human AAA pathology, including VSMC depletion and increased macrophage infiltration. The minimally invasive workflow shortened procedure time and reduced anesthetic exposure, with minimal postoperative pain on veterinary assessment, consistent with limited tissue trauma. Hence, while further investigation is needed, this innovative large-animal model may provide a valuable platform for testing novel endovascular devices and evaluating translational therapeutic strategies.

## Supplementary information

Supplementary information

## Data Availability

The per-animal quantitative datasets used for statistical analysis are provided in the Supplementary Material. Additional raw data generated during this study are available from the corresponding author upon reasonable request.

## Abbreviations

2 W: Two weeks
4 W: Four weeks
AAA: Abdominal aortic aneurysm
BAPN: β-aminopropionitrile
CaCl_2_: Calcium chloride
ECM: Extracellular matrix
Gal-3: Galectin-3
i.v.: Intravenous(ly)
IF: Immunofluorescence
PBS: Phosphate-buffered saline
VSMC: Vascular smooth muscle cells
WB: Western blotting
α-SMA: α-smooth-muscle-actin
