# Supplementary material for: An endovascular porcine model of abdominal aortic aneurysm for interventional radiology research
Source: Eur Radiol Exp. 2026 Jan 26;10:6. doi: 10.1186/s41747-025-00673-z (PMC12834893; doi:10.1186/s41747-025-00673-z)
Supplement: Supplementary file 1 — Supplementary information [file 41747_2025_673_MOESM1_ESM.pdf]

# An Endovascular Porcine Model of Abdominal Aortic Aneurysm for Interventional Radiology Research
# ELECTRONIC SUPPLEMENTARY MATERIAL

## Methods

### Animals

Sample size was set at *n* = 4 for endpoint groups as was statistically feasible and ethically appropriate for a large-animal pilot-study. Three weeks before the intervention, animals arrived at the Research Facilities for Experimental Medicine of the Charité–Universitätsmedizin Berlin to ensure adaptation to researchers and the experimental environment.

### Animal housing specifications

Pigs were acquired in groups of three between February 2023 and February 2024 from the Bundes Hybrid Zuchtprogramm, Dahlenburg-Ellringen, Germany, and Gerd Heinrichs, Heinsberg, Germany.

Upon arrival at the animal facility, the pigs were weighed and clinically examined. They were housed in a fully tiled pen measuring 12.86 $m^2$, which could be divided into two separate pens for training sessions. Straw and sawdust were provided as bedding. The animals were exposed to natural daylight and additional artificial lighting from 6 AM to 6 PM. The ambient temperature was maintained at 20 ± 2 °C, with relative humidity at 55 ± 10%. Water was available ad libitum through two self-drinkers.

The diet consisted of 'Pig feed 1 [>35 kg LM] energy-reduced 4 mm' pellets (Ssniff, Soest, Germany). The animals received 70 g of pellets per kilogram of body weight per day, divided into two meals. Hay was provided as a dietary supplement. During training, food rewards such as banana mash, apples, and canned dog food were used. For enrichment, chains, balls, and rubber toys were permanently available. The enclosure was cleaned daily, with all food leftovers and bedding material replaced.

### Peri-procedural refinements and animal welfare

Procedural safety was enhanced through real-time angiographic leak testing, standardized enzyme/$CaCl_2$ dwell times, and refined balloon-pressure protocols. Post-operative multimodal analgesia (transdermal fentanyl, metamizole, and clinical observation scoring) ensured adequate pain control. Humane endpoints were defined in accordance with Federation of European Laboratory Animal Science Associations–FELASA guidelines [37]. No animal exceeded these criteria after refinement of the induction protocol.

### Ultrasound imaging

Imaging was performed using an ArtUs EXT-2H beamformer with a C5-2H60-A5 transducer (Telemed Ltd., Vilnius, Lithuania), and recordings were acquired via Echo Wave II software (Version 4.2.0, 64-bit). Animals were sedated for baseline and terminal scans, while interim follow-ups were performed in awake animals made possible by the extended period of habituation and acclimatization to research staff prior to the interventional proceedings.

To ensure consistent aortic visualization, ultrasound was conducted with a penetration depth of approximately 120 mm, a dynamic range of 72 dB, a frame rate of 48–50 frames per second, and a transducer frequency of 5 MHz.

In cases where colonic gas interfered with imaging, additional pressure was applied; if visualization remained inadequate, scanning was repeated from the left flank instead of the right.

All *in vivo* aortic diameters were measured outer-to-outer during end-diastole at a standardized site 2 cm below the renal artery branches.

Intra-observer reproducibility of ultrasound diameter measurements was assessed using repeated measurements obtained during standardized image acquisition (three measurements per image) and summarized as the coefficient of variation (CoV) (Table S9)

*Ex vivo* diameters were obtained on unfixed aortic specimens using digital calipers at the corresponding segment. Paraffin-embedded sections were used for qualitative assessment only, and no shrinkage correction was applied, as fixation-related size changes do not affect relative group comparisons.

**Harvest procedure**

The aorta was meticulously dissected from the aortic trifurcation to the suprarenal segment during necropsy, flushed with PBS to clear the lumen, and thereupon excised. The external diameter of the aorta, along with the measurement of the dilation, was recorded using a caliper. As internal control for histopathology, IF and WB analyses, nonaneurysmal suprarenal aortic tissue from the same animals was collected and processed.

Tissue specimens collected from the distal suprarenal aorta and the infrarenal aorta were either embedded in optimal cutting temperature compound and stored at –80°C or fixed in a formaldehyde substitute (MorFFFix®, MORPHISTO GmbH, Offenbach, Germany) for a minimum of 48 hours for histological examination. The formaldehyde-fixed samples were then dehydrated and embedded in paraffin before sectioning. Both optimal cutting temperature- and paraffin-embedded samples were sliced into 10-µm sections and mounted onto adhesive slides for additional analysis. Specimens designated for WB were stored at –20 °C without any additives.

**Histopathology**

The total aortic diameter, luminal and outer circumferences, aortic wall thickness, and total area were meticulously quantified. Measurements were derived from five stitched slide images per aorta specimen and averaged to ensure reproducibility and statistical integrity. For each animal, five stitched histological slides encompassing the entire aortic circumference were analyzed. Quantitative

measurements from these slides were averaged to obtain the per-animal mean, which was then used to compute group means for statistical analysis and graphical representation.

**Immunofluorescence**

Frozen tissue sections were fixed using acetone for 10 min at −20 °C, followed by a tripartite washing protocol in PBS with each wash lasting three min. Primary and secondary antibodies were diluted in background-reducing antibody diluent; the IF staining procedure involved incubating the sections with primary antibody overnight at 4°C in a humidified chamber. Following incubation, the sections were washed three times with PBS for two min each, followed by one-hour of incubation with secondary antibodies, Goat anti-Mouse IgG (H+L) or Donkey anti-Rabbit IgG (H+L) (Highly Cross-Adsorbed Secondary Antibody, Alexa Fluor™ 647, #A-21236, #A-31573, 1:1000; Invitrogen, Thermo Fisher Scientific Inc., Waltham MA, USA). The samples were then rewashed three times in PBS and subsequently mounted with DAPI staining solution.

**Western blotting**

Frozen biological specimens obtained from both infrarenal and suprarenal segments of the aorta were rinsed in PBS and then lysed in a buffer formulation containing 10% sodium dodecyl sulphate, 1M TRIS-HCl, 7M urea, and glycerol. To prevent proteolytic degradation, the buffer solution was supplemented with a protease- and phosphatase-inhibitor blend before homogenization and subsequent incubation at 4 °C for 90 min.

Following the incubation period, the samples underwent centrifugation at $8.05 \times 10^3$ *g* at 4 °C for 20 min. The total protein concentration of the resulting lysates was quantified using a bicinchoninic acid assay. As analysis was performed using the Jess™ Simple Western automated capillary-based size separation and nano-immunoassay system, the protein extracts were then diluted to a final concentration of 1.2 mg/mL using 0.1× Sample Buffer and Fluorescent 5× Master Mix. The lysates were denatured at 95 °C for five min before loading into 12–230 kDa Jess™ separation module plates. Chemiluminescent detection was performed using Jess™ Anti-Rabbit Detection Modules for primary antibody Gal-3 (#14979-1-AP, 1:10; Proteintech Group Inc., Rosemont, IL, USA). Jess™ Anti-Mouse Detection Modules were employed for quantification of α-SMA (#sc-53142, 1:1000; Santa Cruz Biotechnology Inc., Dallas, TX, USA).

To ensure consistency and reliability of data analysis, a Jess™ RePlex™ Module was incorporated in each sample run for total protein detection (#RP-001, #DM-TP01; ProteinSimple™ Biotechne Corp., Minneapolis, MN, USA).

**Sample size and randomization**

The sample size was determined based on prior large-animal AAA studies using comparable endpoints, which typically achieved statistically significant differences in aortic diameter and protein expression with 6−8 animals per group. Fourteen animals were included to account for anticipated early dropouts and to ensure at least eight complete datasets for analysis.

After successful aneurysm induction, animals were randomly assigned to the two-week ($n$ = 4) or four-week ($n$ = 4) experimental endpoint groups using a simple randomization procedure in GraphPad Prism (Version 10.4.1, Dotmatics, Boston, MA, USA), with animal identification numbers (as provided in the experimental animal record sheet) used as the randomization variable.

**Blinding**

Imaging data (angiography, ultrasound) and histological/IF analyses were evaluated blinded to experimental group by one observer. WB quantification was performed in duplicate and analyzed using coded sample identifiers to maintain blinding during data processing.

**Statistical analyses**

The most representative slides were subsequently imaged and analyzed for datasets following a Gaussian distribution, as validated by Shapiro-Wilk tests and Levene's test for equal variances. Parametric tests, such as Tukey's multiple comparison test and one-way ANOVA, were employed. For non-normally distributed data, Brown-Forsythe and Welch ANOVA, as well as Games-Howell's or Dunnett's T3 multiple comparison tests, were utilized when normality was not met. A Pearson correlation analysis was performed to assess the agreement between *in vivo* ultrasound and *ex vivo* caliper-based diameter measurements (Fig. S4a, Table S5).

## Results

**Intra-observer reproducibility of ultrasound diameter measurements**

To assess within-observer repeatability of the ultrasound-based maximal aortic diameter measurements, three independent ROI placements were performed per image during standardized analysis. The coefficient of variation (CoV = SD/mean × 100) was calculated for each animal at the defined endpoint timepoints (2-week: Day 14; 4-week: Day 30; Control: Day 0). CoV values ranged from 2.4–8.0% across all groups (Supplementary Table S9), indicating high measurement consistency under the standardized acquisition and reading workflow.

**Western blot and immunofluorescence analysis of inflammation and vascular remodeling**

To evaluate macrophage expression, vascular remodeling, immune response, and ECM degradation, WB protein analysis was conducted utilizing antibodies targeting the macrophage marker Gal-3 as well as the VSMC biomarker α-SMA (Fig. 6).

WB analysis quantified α-SMA protein expression of 45.97 ± 17.26% (compared to control 80.94 ± 14.26%, $p$ = 0.005, normalized to peak protein expression) for animals subjected to interventional procedures. Comparison of the different time points of analysis (2W *versus* 4W)

reveals that the group euthanized 2W after AAA induction demonstrated a higher preservation of α-SMA (2W: 52.79 ± 14.99% *versus* 39.16 ± 17.52% 4W, $p < 0.001$).

IF analysis confirmed downregulation of α-SMA to 2.04 ± 1.43% (compared to control 35.18 ± 5.15%, $p = 0.003$) in the 4W group, which further substantiates the hypothesis that ECM degradation and smooth muscle cell apoptosis are aligned with the pathogenesis of human abdominal aortic aneurysms following this enzymatic degradation induction model (Fig. 7). The animals examined after 2W exhibited slightly higher α-SMA expression by IF, averaging 3.09 ± 0.7% ($p = 0.003$ compared to control).

Both experimental groups at 2W and 4W post-procedure exhibited upregulated Gal-3 expression, as demonstrated by IF (15.88 ± 4.36%) compared to controls (0.66 ± 0.27%, $p = 0.021$). WB quantification of Gal-3 protein corroborated these trends, showing relative expression levels of 87.22 ± 11.14% (4W) and 46.55 ± 7.31% (2W), compared to 18.86 ± 1.0% in controls ($p < 0.001$).

## Discussion

### Advancement beyond the elastase-only approach by Marinov et al.

Expanding on these efforts, Marinov et al developed a model that used elastase perfusion only, producing significant results regarding histopathological changes in elastic fiber structure and VSMC depletion. However, this model did not achieve a diameter increase greater than 50% compared to the control group [6]. In contrast, our protocol of mechanical stretching followed by active substance application led to a consistently more pronounced infrarenal aortic ectasia, evident as early as 2W post-induction by ultrasound. This approach thus offers a more favorable translational perspective for advancing AAA research opportunities than the model presented by Marinov et al. [6].

### Adaptations to the hybrid technique of Hyneček et al.

Hyneček et al investigated a hybrid approach combining endovascular and surgical techniques to induce infrarenal aneurysms in a porcine model [7]. This involved elastase and collagenase perfusion alongside mechanical stretching, leading to a 73% increase in mean aortic diameters. Significant histological changes were noted in the first week, including endothelial denudation, neutrophil infiltration, and elastin degradation, as well as a consistent decrease in smooth muscle cell population observed at three and six weeks [7]. They also recommended ligating the lumbar arteries to prevent systemic flushing of elastase and collagenase, which we chose not to implement. We observed that excluding these arteries from circulation resulted in an increased risk of paralysis post-intervention. Therefore, we modified our balloon catheter placement to render the closure of those branches unnecessary. This adjustment minimized ischemia risk, reduced the need for additional costly interventional materials such as coils, and shortened procedure time.

**Vascular inflammation and extracellular matrix component degeneration**

Recent studies show that adding collagenase as an additional enzymatic agent better replicates the ECM profile of human AAA, supporting its use in translational models [27, 33]. In line with these reports, our data confirm that mechanical dilation followed by intraluminal application of elastase, collagenase, and $CaCl_2$, induces the formation of large aneurysms, with ultrasound revealing a statistically significant increase in aortic diameter by the second week. Despite the rapid growth, the model preserves key features of human aortic wall degeneration such as matrix-metalloproteinase-2 macrophage activation as a response to inflammatory aortic wall destruction [34].

**Limitations**

Although the study was conducted in young female swine, prior work indicates that the molecular and structural mechanisms of aneurysm formation (matrix degradation, inflammatory infiltration, and smooth muscle cell loss) are conserved across sex and age in large-animal models [15, 17]. The uniform use of young females reduced variability in vessel size and peri-procedural risk, supporting reproducibility for this feasibility phase. Therefore, while sex and age effects may modulate absolute wall mechanics or aneurysm growth rate, they are unlikely to affect the mechanistic validity of the induction approach presented here.

**Reporting**

**ARRIVE Guidelines: Essential 10**

**1. Study design**

Groups: AAA induction cohort with terminal imaging/harvest at 2W ($n$ = 4) and 4W ($n$ = 4). No separate sham group; suprarenal tissue from the same animals served as internal healthy controls for histology/IF/WB.

Experimental unit: individual swine; held in groups of three.

Objective: establish and evaluate a purely endovascular porcine AAA model using balloon dilation plus intraluminal collagenase, elastase, and $CaCl_2$.

Endpoints: *in vivo* ultrasound diameter trajectory; terminal diameter; histopathology (elastin/collagen fragmentation, calcification); IF (Gal-3, α-SMA); western blot (Gal-3, α-SMA).

**2. Sample size**

Enrolled: $n$ = 14 female German Landrace pigs (30–40 kg).

Completed to endpoints: $n$ = 8 (2W $n$ = 4; 4W $n$ = 4).

Rationale: feasibility/ethics for a large-animal pilot; $n$ = 4 per endpoint group judged appropriate for exploratory work and ethical animal testing following Charité – 3R principles (Replacement, Reduction, Refinement).

**3. Inclusion and exclusion criteria**

Inclusion: clinically healthy female German Landrace, 30–40 kg at intervention.

Humane endpoints/exclusions applied: aortic rupture (*n* = 2); hindlimb paresis after accidental systemic enzyme/$CaCl_2$ flushing due to catheter malfunction (*n* = 3); failed aneurysm induction due to material failure (*n* = 1).
Analysis sets: ultrasound growth and terminal/*ex vivo* assessments in animals reaching 2W or 4W endpoints; internal suprarenal tissue used as control within the same animals.

**4. Randomization**
Animals that completed the protocol were randomly allocated to 2W vs 4W terminal cohorts (*n* = 4 each).
Allocation was performed at the level of individual animals after arrival. Pigs were housed in social groups of three per pen; where vendors indicated potential littermates, we kept presumed siblings together within a pen when feasible, but sibling status was not guaranteed and did not determine group assignment.

**5. Blinding**
**During procedures**: Not blinded (operators must know allocation for dosing/device use).
**Outcome assessment**: Histopathology/IF/WB quantification used predefined thresholds/normalization; analysts were blinded.
**Data analysis**: Statistician aware of group labels.

**6. Outcome measures**
Primary: Aortic diameter progression by weekly ultrasound; terminal diameter confirmation on paraffin sections and gross caliper at necropsy.
Secondary: Histology (elastin, collagen, calcium; morphometrics); IF area fractions for Gal-3 and α-SMA; Western blot densitometry for Gal-3 and α-SMA; feasibility/adverse events.

**7. Statistical methods**
Software: IBM SPSS 29.0; GraphPad Prism 10.4.1.
Data presentation: mean ± SD; $\alpha < 0.05$; 95% CIs where applicable.
Assumptions: Shapiro-Wilk for normality; Levene's for variance homogeneity.
Tests: For normal data, one-way ANOVA with Tukey or Dunnett's T3; for non-normal/heteroscedastic data, Brown-Forsythe/Welch ANOVA with Games-Howell or Dunnett's T3.
Graphs: bar charts with individual points overlaid.

**8. Experimental animals**
Species/strain: *Sus scrofa domestica*, German Landrace.
Sex/age/weight: female; ~12 weeks; 30–40 kg.
Health status & provenance: Clinically examined on arrival; vendors listed (Bundes Hybrid Zuchtprogramm; Gerd Heinrichs); no genetic modification status.

Previous procedures: None.

## 9. Experimental procedures

Facility: Charité – Universitätsmedizin Berlin Research Facilities for Experimental Medicine (FEM); hybrid operating room.

Anesthesia/monitoring: multi-agent induction (atropine, azaperone, ketamine, xylazine), propofol, fentanyl; maintenance with isoflurane and midazolam; intubation and mechanical ventilation; invasive blood pressure monitoring via carotid line; continuous ECG/$SpO_2$/temperature; acid–base and ACT/blood gases at ≤ 30 min intervals; vasoactive support as needed (noradrenaline).

Vascular access: 6F right carotid sheath; 8F right femoral sheath; baseline aortography.

Induction protocol: balloon dilation 14 × 40 mm at 6–8 atm for 10 min (+ ~30% diameter increase target); Fogarty occlusion between first two lumbar pairs; enzymatic incubation collagenase 6000 IU + elastase 500 IU for 20 min; saline flush; reperfusion ten min; repeat occlusion; $CaCl_2$ 25% 0.5 mL for 15 min; flush; closure with Angio-Seal and skin sutures.

Peri-/post-op care: buprenorphine i.v. 0.03 mL/kg; fentanyl patch 50 µg/h for six days; sulbactam/ampicillin and metronidazole i.v.; metamizole i.m. then oral for one week; intensive clinical surveillance including night monitoring.

Ultrasound: baseline under sedation; weekly follow-up in awake habituated animals; standardized acquisition; three images averaged per time point.

Euthanasia and tissue harvest: at 2W or 4W under deep propofol, then pancuronium and potassium chloride; infrarenal and suprarenal segments processed for histology/IF/WB; morphometrics from five stitched images per specimen.

IF: Gal-3 and α-SMA staining on 10 µm cryosections, area fraction quantification.

WB: Jess Simple Western; α-SMA and Gal-3 normalized to peak protein expression during run.

## 10. Results

### Ultrasound diameter

⇒ Day 7, 1.32 ± 0.08 cm ($p < 0.001$).

⇒ Day 14, 1.59 ± 0.06 cm ($p < 0.001$).

⇒ Day 21, 1.81 ± 0.11 cm ($p < 0.001$).

⇒ Day 30, 1.94 ± 0.19 cm ($p = 0.002$).

⇒ Baseline 0.74 ± 0.08 cm.

### Histopathology

Calcification:

⇒ Two weeks 6.73 ± 1.94% ($p = 0.041$ *versus* 4W).

⇒ Four weeks 8.44 ± 2.01% ($p = 0.041$ *versus* 2W).

Elastic fiber fragmentation:

⇒ Two weeks 15.37 ± 3.15% ($p = 0.049$).

⇒ Four weeks 27.87 ± 7.52% ($p = 0.040$).

⇒ Control 9.10 ± 2.17%.

Collagen fragmentation:

⇒ Two weeks 15.97 ± 3.38% ($p = 0.025$).

⇒ Four weeks 24.84 ± 4.75% ($p = 0.014$).

⇒ Control 7.58 ± 1.32%.

**Immunofluorescence**

Gal-3 positive-cell% of aortic area:

⇒ Two weeks: 15.12 ± 3.88% ($p = 0.012$).

⇒ Four weeks: 16.65 ± 5.27% ($p = 0.021$).

⇒ Control: 0.66 ± 0.27%.

α-SMA positive-cell% of aortic area:

⇒ Two weeks 3.09 ± 0.70% ($p = 0.003$).

⇒ Four weeks: 2.04 ± 1.43% ($p = 0.003$).

⇒ Control: 35.18 ± 5.15%.

**Western blot**

α-SMA protein expression:

⇒ Two weeks 52.79 ± 14.99% ($p = 0.005$).

⇒ Four weeks 39.16 ± 17.52% ($p < 0.001$).

⇒ Control 80.94 ± 14.26%.

Gal-3 protein expression:

⇒ Two weeks 46.55 ± 7.31% ($p < 0.001$).

⇒ Four weeks 87.22 ± 11.14% ($p < 0.001$).

⇒ Control 18.86 ± 1.0%.

Adverse events: rupture $n = 2$; hindlimb paresis $n = 3$; non-responder $n = 1$. Events occurred during early protocol development; after standardization, no further such events were observed.

**ARRIVE Guidelines: Recommended Set**

**11. Abstract**

Provides background, aim, methods (endovascular induction with enzymes/$CaCl_2$; ultrasound tracking; *ex vivo* analyses), key results (diameter growth; Gal-3 up; α-SMA down; ECM fragmentation/calcification), and conclusions about feasibility and translational utility.

**12. Background**

Justifies need for large-animal AAA models; limitations of open surgical approaches; value of a minimally invasive endovascular model for translational device/drug testing.

**13. Objectives**

Research question: can a purely endovascular porcine protocol reproducibly induce infrarenal AAA that recapitulates human molecular and structural hallmarks.

Hypotheses: protocol yields progressive diameter enlargement; shows ECM degradation (elastin/collagen fragmentation, calcification), macrophage upregulation (Gal-3), and VSMC depletion (α-SMA); workflow is feasible with manageable morbidity after standardization.

**14. Ethical statement**

All procedures were approved by the State Office for Health and Social Affairs Berlin (LaGeSo) and conducted in accordance with FELASA and ARRIVE guidelines and the Charité – 3R principles (Replacement, Reduction, Refinement). Under registration number G 0077/21, approval was granted on 05 November 2021.

**15. Housing and husbandry**

Pigs were housed in groups of three per pen (12.86 $m^2$; tiled; straw/sawdust bedding) throughout acclimation and the study. Enrichment (chains, balls, rubber toys) was available continuously. Natural daylight plus artificial lighting 06:00 – 18:00; temperature 20 ± 2 °C, humidity 55 ± 10%. Water ad libitum via self-drinkers; Ssniff pellets 70 g/kg/day in two meals with hay; food rewards used during training. Pens were cleaned daily with replacement of leftovers and bedding. Where possible, presumed sibling animals were housed together, but siblings were not always available/confirmed.

**16. Animal care and monitoring**

Refinements include a fully endovascular approach (no laparotomy), multimodal anesthesia/analgesia, vascular closure devices, intensive perioperative monitoring, and habituation enabling awake ultrasound follow-up. Veterinary assessments indicated minimal postoperative pain with no escalation of analgesia required.

**17. Interpretation/scientific implications**

Model achieved large, progressive AAA and recapitulated key human-like molecular and structural changes, supporting use for translational evaluation of endovascular devices and ECM/inflammation-targeted therapies.

**18. Generalizability/translation**

Swine anatomy and vessel size support procedural realism; purely interventional workflow mirrors clinical endovascular practice and facilitates serial assessments.

**19. Protocol registration**

A pre-specified experimental protocol (research question, design, and analysis plan) was prepared before the study and registered/approved with LaGeSo under G 0077/22 (State Office for Health and Social Affairs Berlin) on 05 November 2021.

**20. Data access**

Original datasets available upon reasonable request.

**21. Declaration of interests**

Authors report no competing interests.

## Supplementary tables

**Table S1** Two-way ANOVA results for ultrasound diameter growth

| Comparison | Mean Difference (cm) | 95% CI of Difference | Adjusted *p*-value | Significance |
|---|---|---|---|---|
| **Baseline vs Day 7** | –0.584 | –0.702 to –0.466 | < 0.001 | *** |
| **Baseline vs Day 14** | –0.854 | –0.972 to –0.736 | < 0.001 | *** |
| **Baseline vs Day 21** | –1.023 | –1.175 to –0.870 | < 0.001 | *** |
| **Baseline vs Day 30** | –1.196 | –1.348 to –1.043 | < 0.001 | *** |
| **Day 7 vs Day 14** | –0.270 | –0.388 to –0.152 | < 0.001 | *** |
| **Day 7 vs Day 21** | –0.439 | –0.591 to –0.286 | < 0.001 | *** |
| **Day 7 vs Day 30** | –0.612 | –0.764 to –0.459 | < 0.001 | *** |
| **Day 14 vs Day 21** | –0.169 | –0.321 to –0.016 | 0.026 | * |
| **Day 14 vs Day 30** | –0.342 | –0.494 to –0.189 | < 0.001 | *** |
| **Day 21 vs Day 30** | –0.173 | –0.340 to –0.006 | 0.040 | * |

Pairwise comparisons for the row factor (time) are shown as mean differences in diameter (cm) with 95% CI and adjusted *p*-values. Significance is indicated as follows: $p < 0.05$ (*), $p < 0.001$ (***).
Two-way ANOVA: Row Factor (time) $F(4, 20) = 201.1$, $p < 0.001$; Column Factor (group) $F(7, 20) = 2.84$, $p = 0.031$.
Residual homogeneity verified (Spearman $p = 0.12$); normality verified (Shapiro-Wilk $p = 0.60$).
CI, confidence intervals.

**Table S2** Standardized effect sizes (Hedges' g) with 95% confidence intervals for primary endpoints

| **Endpoint** | **Week 2 (Mean ± SD, *n* = 4)** | **Week 4 (Mean ± SD, *n* = 4)** | Hedges' *g* | 95% CI (*g*) |
|---|---|---|---|---|
| Diameter (cm) | 1.46 ± 0.10 | 1.94 ± 0.20 | **2.64** | **0.43 to 4.85** |
| Galectin-3 (% area) | 15.12 ± 3.88 | 16.65 ± 5.27 | **0.29** | **–1.11 to 1.68** |
| α-smooth muscle actin (% area) | 3.09 ± 0.70 | 2.04 ± 1.43 | **–0.82** | **–2.30 to 0.67** |
| Elastin (% area) | 15.37 ± 3.15 | 27.87 ± 7.52 | **1.89** | **0.03 to 3.74** |
| Collagen (% area) | 15.97 ± 3.38 | 24.84 ± 4.75 | **1.87** | **0.03 to 3.72** |
| Calcium (% area) | 6.73 ± 1.94 | 8.44 ± 2.01 | **0.75** | **–0.72 to 2.22** |
| α-smooth muscle actin (Western Blot) | 12 683 986.65 ± 1 340 263.54 | 6 684 553.20 ± 687 516.93 | **−4.90** | **−8.37 to −1.42** |
| Galectin-3 (Western Blot) | 5 955 432.23 ± 647 762.91 | 11 720 438.18 ± 543 461.74 | **8.38** | **2.76 to 14.01** |

Effect sizes were calculated using the pooled standard deviation with small-sample correction. Confidence intervals were derived from the standard error of *d* using a normal approximation. Positive *g* values reflect higher values in the Week-4 group; negative values reflect lower values in the Week-4 group, as expected for α-smooth muscle actin due to progressive smooth muscle cell loss.

**Table S3** Linear mixed-effects model results for longitudinal ultrasound aortic diameter measurements

| Component / Effect | Statistic | Value |
|---|---|---|
| **Effect of Time (fixed)** | $F(4,20)$ | 201.07 |
| | *p*-value | < 0.001 |
| **Effect of Animal (random intercept)** | $F(7,20)$ | 2.84 |
| | *p*-value | 0.031 |
| **Variance explained by Time** | | 84.44% |
| **Variance explained by Animal** | | 2.09% |
| **Random-intercept variance** | | 0.01771 |
| **Residual variance** | | 0.006233 |

A mixed-effects model was used to account for repeated measurements within the same animal; model specification: Diameter ~ Time + (1 | Animal). Time was modeled as a fixed effect and Animal as a random intercept term. The table reports *F*-statistics, associated degrees of freedom ($DF_n$, $DF_d$), *p*-values, and variance components. Variance explained corresponds to the proportion of total variability attributable to each model component. Random-intercept variance reflects between-animal variability after accounting for the effect of Time; residual variance represents within-animal measurement variability.

**Table S4** Model assumption checks for ultrasound diameter ANOVA

| Test | Statistic | *p*-Value | Result |
|---|---|---|---|
| **Shapiro-Wilk (W)** | 0.973 | 0.599 | Passed *(normality)* |
| **D'Agostino-Pearson ($K^2$)** | 1.248 | 0.536 | Passed *(normality)* |
| **Anderson-Darling ($A^2$)*** | 0.313 | 0.531 | Passed *(normality)* |
| **Kolmogorov-Smirnov (distance)** | 0.121 | 0.100 | Passed *(normality)* |
| **Spearman rank correlation (residuals)** | 0.210 | 0.124 | Passed *(homoscedasticity)* |

Normality was confirmed by Shapiro-Wilk, D'Agostino-Pearson, Anderson-Darling, and Kolmogorov-Smirnov tests. Homoscedasticity (equal residual variance) was verified by Spearman rank correlation between predicted and absolute residuals ($p$ = 0.124). All tests indicated that ANOVA assumptions were satisfied.

**Table S5** Correlation between *in vivo* and *ex vivo* diameter measurements

| Variable 1 | Variable 2 | *n* | Pearson *r* | *p*-Value |
|---|---|---|---|---|
| ***In vivo* diameter (cm)** | ***Ex vivo* diameter (cm)** | 8 | 0.9999 | < 0.001 |

Pearson correlation analysis demonstrated excellent agreement between ultrasound-derived diameters and unfixed caliper measurements ($r$ = 0.9999, $p$ < 0.001, $n$ = 8). This confirms consistency between imaging-based and macroscopic assessments of aneurysm size.

**Table S6** Dual-occlusion procedural safety checklist for endovascular aneurysm induction

| Step | Procedure | Verification | Purpose |
|---|---|---|---|
| 1 | **Positioning of proximal and distal balloons** (Fogarty® Arterial Embolectomy Catheters, 5.5F/6F; Edwards Lifesciences Corp.) at level of 3rd/4th lumbar artery under fluoroscopy | Fluoroscopic confirmation of tip location | Defines isolated infrarenal segment for enzyme/$CaCl_2$ dwell |
| 2 | **Balloon inflation** with diluted contrast under pressure monitoring | Gradual inflation until luminal sealing; verify no contrast leak proximally or distally | Ensures complete segmental isolation and prevents reflux |
| 3 | **Leak check** by gentle angiographic contrast injection through central microcatheter (Progreat™, Terumo Medical Corp.) | Visualization of confined contrast column between balloons | Confirms correct sealing before enzyme/$CaCl_2$ infusion |
| 4 | **Infusion of enzyme/$CaCl_2$ solution** into the isolated segment | Real-time visualization of filling and stable pressure curve | Ensures uniform exposure without systemic spill |
| 5 | **Incubation period/dwell time** | Timed: enzyme 20 min; $CaCl_2$ 15 min | Achieves controlled degradation while minimizing ischemia |
| 6 | **Negative pressure aspiration** | Visible withdrawal of opaque fluid under fluoroscopy | Removes residual enzyme/$CaCl_2$ to avoid systemic distribution |
| 7 | **Balloon deflation** (distal then proximal) | Monitor for reflow under fluoroscopy; confirm distal perfusion | Controlled reperfusion and avoidance of embolic release |
| 8 | **Final angiographic run** | Visualization of patent lumbar and renal branches; no extravasation | Confirms restoration of flow and procedural safety |

Stepwise overview of the dual-balloon occlusion protocol performed under fluoroscopic and angiographic guidance. The table summarizes the sequential positioning, inflation, leak-testing, incubation, and reperfusion steps used to ensure procedural safety and segmental confinement of

the enzyme/$CaCl_2$ solution. Occlusion times were standardized to approximately 20 min for enzymatic incubation and 15 min for $CaCl_2$ dwell. Continuous balloon pressure monitoring was applied throughout the procedure to prevent systemic leakage and to confirm complete restoration of flow before reperfusion.

**Table S7.** Absolute western blot signal intensity values for α-SMA and Galectin-3

| Protein | Group | *n* | Mean (PE units) | SD | SEM | Median |
|---|---|---|---|---|---|---|
| α-smooth-muscle-actin | Week 2 | 4 | 12 683 986.7 | 1 340 263.5 | 670 131.8 | 12 553 005.1 |
| | Week 4 | 4 | 6 684 553.2 | 687 516.9 | 343 758.5 | 6 422 665.5 |
| | Control | 4 | 22 074 849.5 | 1 741 459.5 | 870 729.7 | 21 982 053.9 |
| Galectin-3 | Week 2 | 4 | 5 955 432.2 | 647 762.9 | 323 881.4 | 5 900 766.4 |
| | Week 4 | 4 | 11 720 437.6 | 543 461.7 | 271 730.9 | 11 816 859.5 |
| | Control | 4 | 1 779 879.6 | 105 216.0 | 52 608.0 | 1 815 786.6 |

Raw (non-normalized) protein expression values derived from Jess capillary western blot analysis. Data are reported in arbitrary PE units as provided by the system output. Mean, SD, SEM, and median, are shown for each experimental group and suprarenal control tissue (*n* = 4 animal's samples per group). These data served as the basis for normalized relative expression (100% scale) used in the main analysis and presented in *Fig. 6*. Statistical comparisons were performed on normalized values as described in the Statistical Analyses section.
α-SMA, α-smooth-muscle-actin; PE, protein expression; SD, standard deviation; SEM, standard error of mean.

**Table S8** *Post hoc* precision analysis for primary endpoints

| Endpoint | Group (*n*) | Mean ± SD | 95% CI of Mean | CI Width | Comment |
|---|---|---|---|---|---|
| **Diameter (cm)** | 2 weeks (4) | 1.46 ± 0.04 | 1.38 – 1.54 | ± 0.08 | High precision |
| | 4 weeks (4) | 1.94 ± 0.20 | 1.75 – 2.13 | ± 0.19 | Adequate precision |
| **Galectin-3 (%)** | 2 weeks (4) | 54 ± 2 | 52 – 56 | ± 2 | Tight CI |
| | 4 weeks (4) | 91 ± 8 | 83 – 99 | ± 8 | Good precision |
| **α-smooth muscle actin (%)** | 2 weeks (4) | 53 ± 6 | 47 – 59 | ± 6 | Moderate precision |
| | 4 weeks (4) | 28 ± 3 | 25 – 31 | ± 3 | Good precision |

Mean ± SD, 95% confidence intervals, and CI widths are provided for key outcomes at two- and four-week endpoints. Ultrasound, Galectin-3, and α-smooth-muscle-actin results demonstrate narrow confidence intervals relative to effect size, confirming adequate precision of estimates. CI, Confidence interval..

**Table S9** Intra-observer reproducibility of ultrasound maximal aortic diameter measurements

| Group and endpoint | Mean CoV (%) | Range (%) | Notes |
|---|---|---|---|
| 2 weeks (Day 14; *n* = 4) | **5.8%** | 2.7–8.0% | Early aneurysm formation |
| 4 weeks (Day 30; *n* = 4) | **5.0%** | 2.4–8.0% | Advanced aneurysm expansion |
| Control (Day 0; *n* = 4) | **5.0%** | 2.6–6.8% | Normal-caliber aorta |

Maximal aortic diameter was measured in three ultrasound images per timepoint and per animal as part of the standardized reading workflow. Reproducibility was quantified using the coefficient of variation (CoV = SD/mean × 100). Reported values represent the group mean CoV and range across animals at the designated endpoint timepoints. Lower CoV indicates higher within-observer measurement consistency.

CoV values remained low across all groups, reflecting stable and repeatable diameter assessment under the standardized acquisition and image analysis protocol.

**Table S10** Per-animal dataset for key quantitative outcome measures

| Animal **ID** | Group | *In vivo* D (cm) | *Ex vivo* D (cm) | α-SMA IF (%) | α-SMA WB (PEU) | Gal-3 IF (%) | Gal-3 WB (PEU) | Elastin Share (%) | Collagen Share (%) | Calcium Share (%) |
|---|---|---|---|---|---|---|---|---|---|---|
| **A-1** | Week 2 | 1.65 | 1.47 | 3.38 | $1.284 \times 10^7$ | 19.16 | $5.38 \times 10^6$ | 12.85 | 14.21 | 5.33 |
| **A-2** | Week 2 | 1.58 | 1.44 | 2.11 | $1.442 \times 10^7$ | 10.45 | $5.42 \times 10^6$ | 19.65 | 19.88 | 9.60 |
| **A-3** | Week 2 | 1.57 | 1.43 | 3.15 | $1.121 \times 10^7$ | 13.59 | $6.64 \times 10^6$ | 15.84 | 17.48 | 5.84 |
| **A-4** | Week 2 | 1.67 | 1.51 | 3.73 | $1.226 \times 10^7$ | 17.28 | $6.38 \times 10^6$ | 13.15 | 12.29 | 6.13 |
| **A-5** | Week 4 | 2.21 | 1.84 | 2.99 | $6.59 \times 10^6$ | 18.03 | $1.21 \times 10^7$ | 28.67 | 29.79 | 7.23 |
| **A-6** | Week 4 | 1.84 | 1.81 | 1.15 | $6.21 \times 10^6$ | 18.84 | $1.22 \times 10^7$ | 30.06 | 19.27 | 8.51 |
| **A-7** | Week 4 | 1.77 | 1.58 | 3.49 | $7.68 \times 10^6$ | 8.93 | $1.10 \times 10^7$ | 17.44 | 22.68 | 11.25 |
| **A-8** | Week 4 | 1.94 | 1.83 | 0.51 | $6.26 \times 10^6$ | 20.78 | $1.16 \times 10^7$ | 35.30 | 27.60 | 6.77 |
| **C-1** | Control | 0.87 | 0.78 | 36.18 | $2.31 \times 10^7$ | 0.65 | $1.82 \times 10^6$ | 9.71 | 8.45 | 0 |
| **C-2** | Control | 0.78 | 0.75 | 30.01 | $2.09 \times 10^7$ | 0.33 | $1.81 \times 10^6$ | 10.82 | 8.57 | 0 |
| **C-3** | Control | 0.73 | 0.69 | 32.62 | $2.40 \times 10^7$ | 0.66 | $1.86 \times 10^6$ | 9.93 | 7.60 | 0 |
| **C-4** | Control | 0.72 | 0.72 | 41.91 | $2.04 \times 10^7$ | 0.99 | $1.63 \times 10^6$ | 5.93 | 5.71 | 0 |

This table provides per-animal quantitative values for all primary morphological, molecular, and histological endpoints, including maximal aortic diameter (*in vivo* and *ex vivo*), α-smooth muscle actin (α-SMA) and Galectin-3 (Gal-3) expression (immunofluorescence and Western blot), and elastin, collagen, and calcium content (all reported as % aortic area). Week 2 animals were assessed at Day 14 and Week 4 animals at Day 30. Control animals were assessed at baseline (Day 0) for *in vivo* diameter, and *ex vivo* measurements were obtained from the suprarenal aorta. Providing the dataset at per-animal resolution supports reproducibility and enables independent re-analysis of the reported findings.

**Supplementary figures**

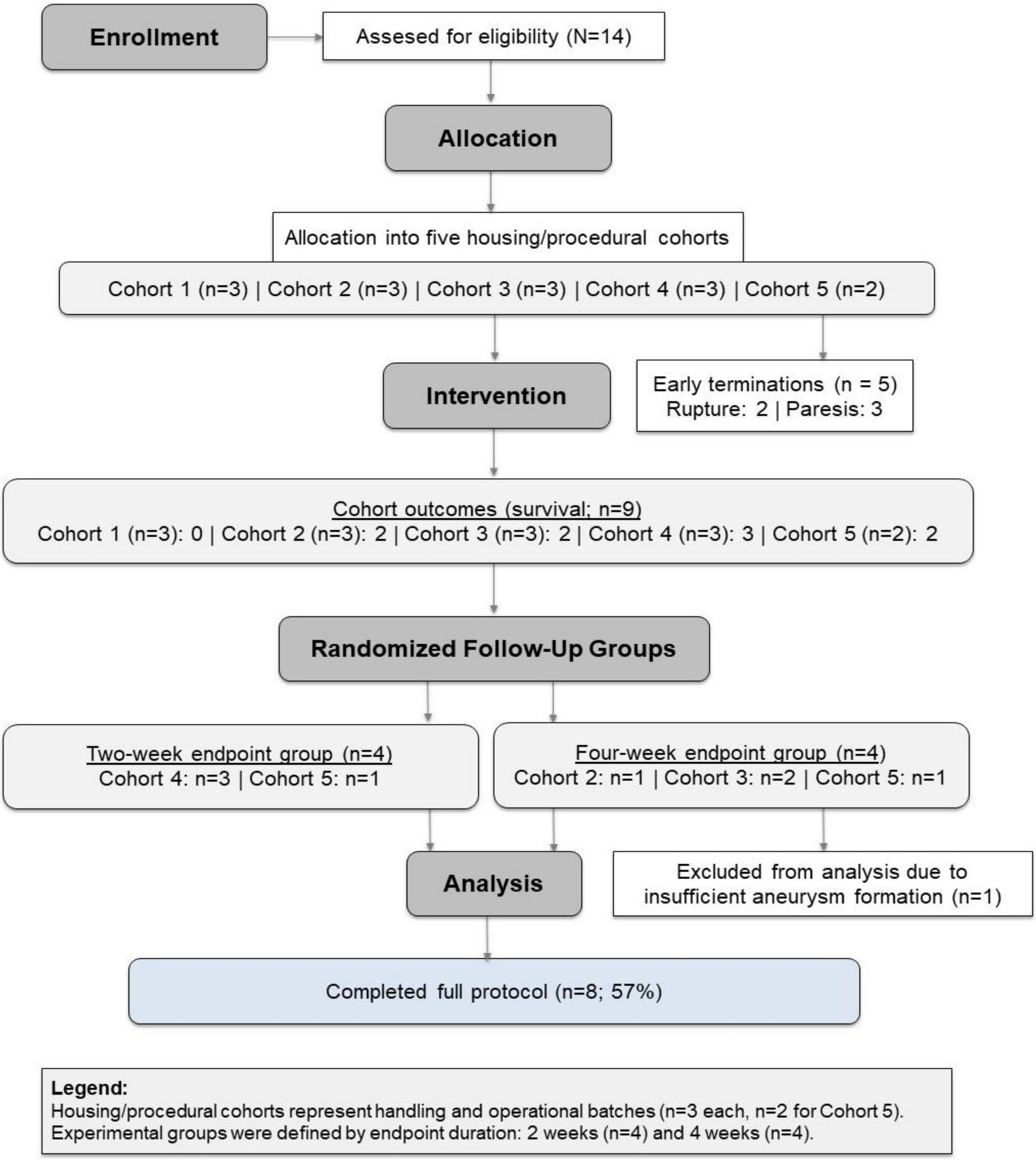


**Fig. S1** CONSORT-style flow diagram for animal allocation, complications, and outcomes in the porcine abdominal aortic aneurysm model. Animals were organized into five housing and procedural cohorts (batches of *n* = 3/*n* = 2 in cohort 5, each) within the planned experimental design. Early complications occurred mainly in the first three cohorts, while all animals in cohorts 4 and 5 completed the study without complications. Eight animals successfully reached the planned study endpoints defined by observation periods: a two-week endpoint group (*n* = 4) and a four-week endpoint group (*n* = 4). CONSORT, Consolidated Standards of Reporting Trials.

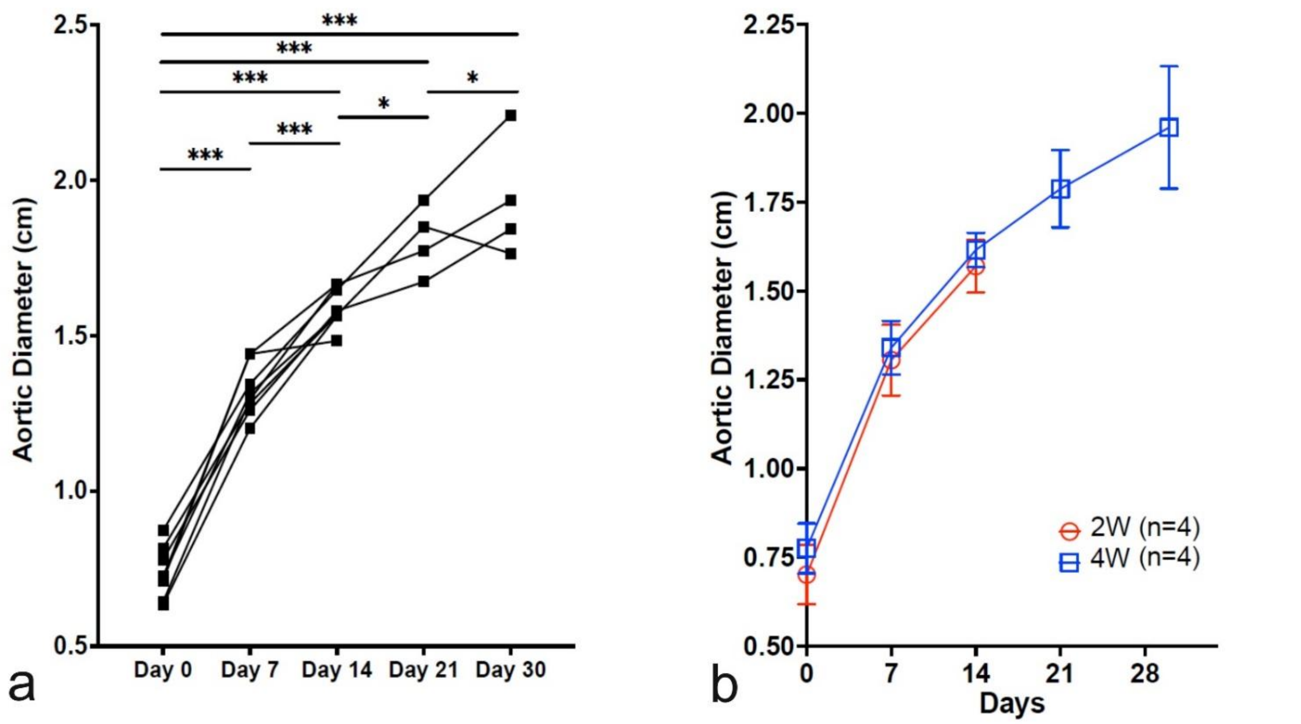


| Comparison | Mean Difference (cm) | 95 % CI of Difference | Adjusted p-value | Significance |
|---|---|---|---|---|
| **Baseline vs Day 7** | −0.584 | −0.702 to −0.466 | < 0.001 | *** |
| **Baseline vs Day 14** | −0.854 | −0.972 to −0.736 | < 0.001 | *** |
| **Baseline vs Day 21** | −1.023 | −1.175 to −0.870 | < 0.001 | *** |
| **Baseline vs Day 30** | −1.196 | −1.348 to −1.043 | < 0.001 | *** |
| **Day 7 vs Day 14** | −0.270 | −0.388 to −0.152 | < 0.001 | *** |
| **Day 14 vs Day 21** | −0.169 | −0.321 to −0.016 | 0.026 | * |
| **Day 21 vs Day 30** | −0.173 | −0.340 to −0.006 | 0.040 | * |

c

**Fig. S2** Longitudinal ultrasound-based aortic diameter development. (**a**) Individual "spaghetti" trajectories showing outer-to-outer aortic diameter measurements for each animal across time points (Baseline, Days 7, 14, 21, 30). Lines connect repeated measurements within the same animal; horizontal bars indicate statistically significant differences between time points. Statistical significance: $p < 0.05$ *, $p < 0.001$ ***; Tukey's post-hoc, $n = 4$ day 0–day 14, $n = 4$, day 14–day 30). (**b**) Grouped mean ± SD line plot derived from the two-way ANOVA (time × group). Progressive diameter enlargement was observed with a significant main effect of time ($F(4, 20) = 201.1$, $p < 0.001$) and a modest group effect ($F(7, 20) = 2.84$, $p = 0.031$). Error bars represent SD; $n = 4$ per endpoint group. (**c**) Summary of Tukey's multiple-comparison results showing mean differences, 95% confidence intervals, adjusted $p$-values, and significance levels for all time-point contrasts. Model assumptions were fulfilled (Shapiro-Wilk $p = 0.60$; Spearman test for heteroscedasticity $p = 0.12$).

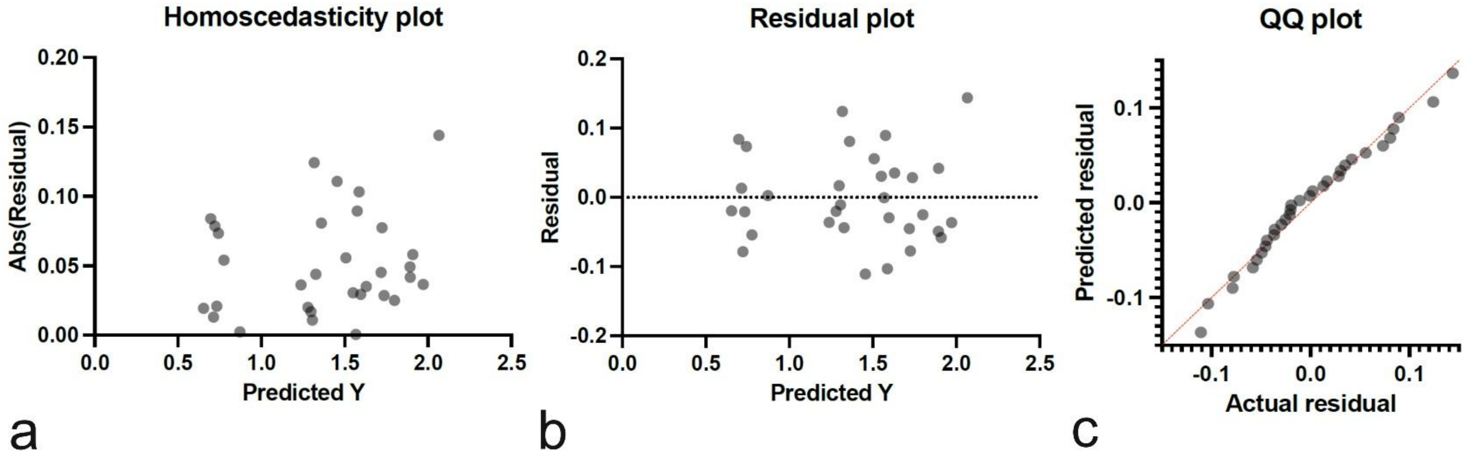


**Fig. S3.** Diagnostic plots for ultrasound diameter two-way ANOVA. (**a**) Homoscedasticity plot showing absolute residuals versus predicted values. No trend was observed (Spearman $r = 0.21$, $p = 0.12$), confirming variance homogeneity. (**b**) Residuals versus predicted values demonstrating random scatter around zero, indicating absence of systematic bias. (**c**) Quantile-quantile (*QQ*) plot confirming normally distributed residuals (Shapiro-Wilk $p = 0.60$). All diagnostic tests passed, supporting the validity of the ANOVA model assumptions. *QQ* Quantile-

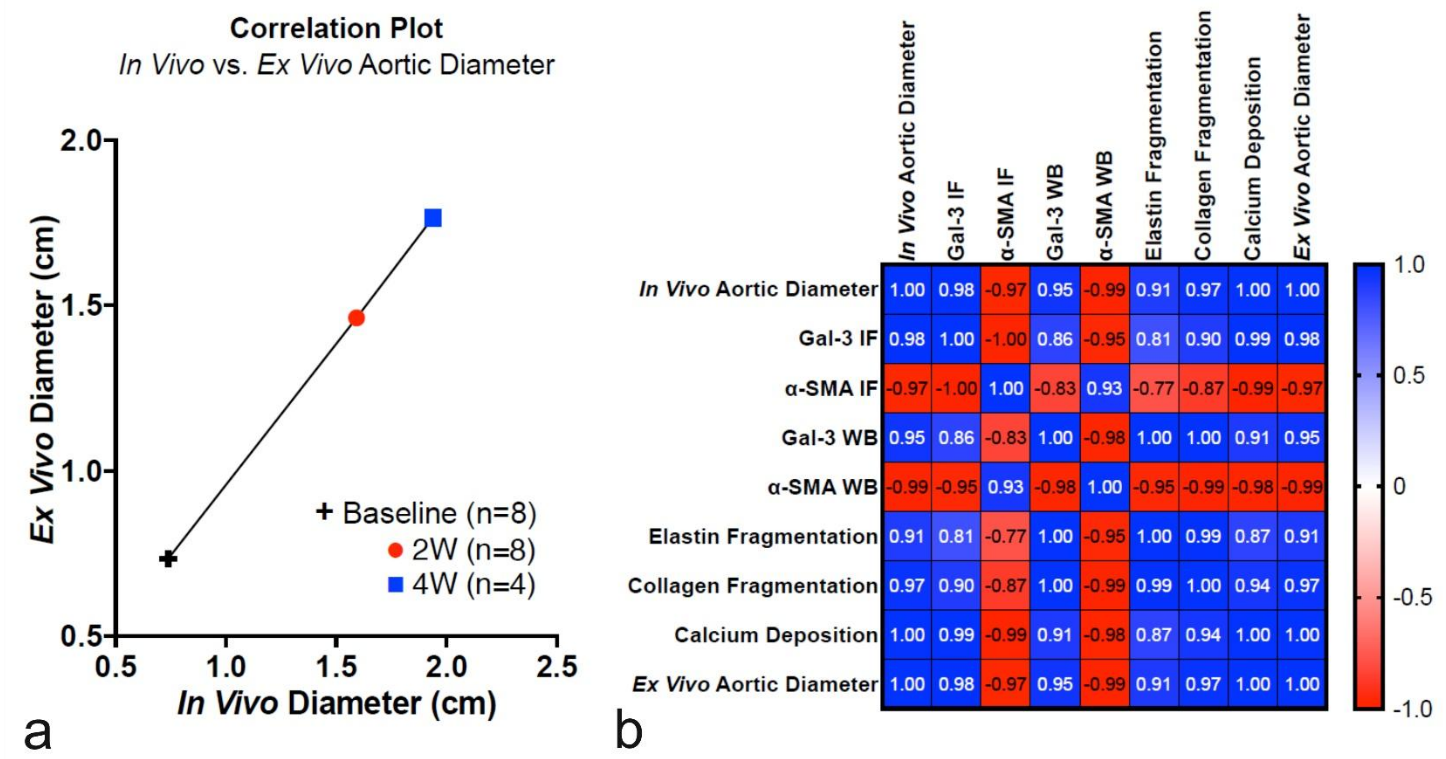


**Fig. S4** Imaging-pathology correlations and validation of ultrasound measurements. (**a**) *In vivo* ultrasound versus *ex vivo* caliper measurements of infrarenal aortic diameter. Each point represents an individual animal (baseline: *black cross*; two weeks: *red circle*; four weeks: *blue square*). A strong linear relationship was observed ($r = 0.999$, $p < 0.001$), confirming consistency between ultrasound and direct measurement.

(**b**) Pearson correlation heatmap illustrating the relationships between in vivo aortic diameter and histopathological or molecular markers of remodeling. Ultrasound-derived diameters correlated positively with Galectin-3 immunofluorescence (*Gal-3 IF,* $r = 0.98$) and western blot expression (*Gal-3 WB,* $r = 0.95$), and with elastic and collagen fiber fragmentation ($r = 0.91$–$0.97$). In contrast, α-smooth-muscle-actin (*α-SMA IF/WB*) showed strong inverse correlations ($r = -0.95$ to $-0.99$), indicating smooth-muscle loss with increasing aneurysm size. All correlations were significant ($p < 0.01$). The color scale denotes correlation strength (*blue = positive, red = negative*). *α-SMA* α-smooth-muscle-actin, *Gal-3* Galectin-3, *IF* Immunofluorescence, *WB* Western blot

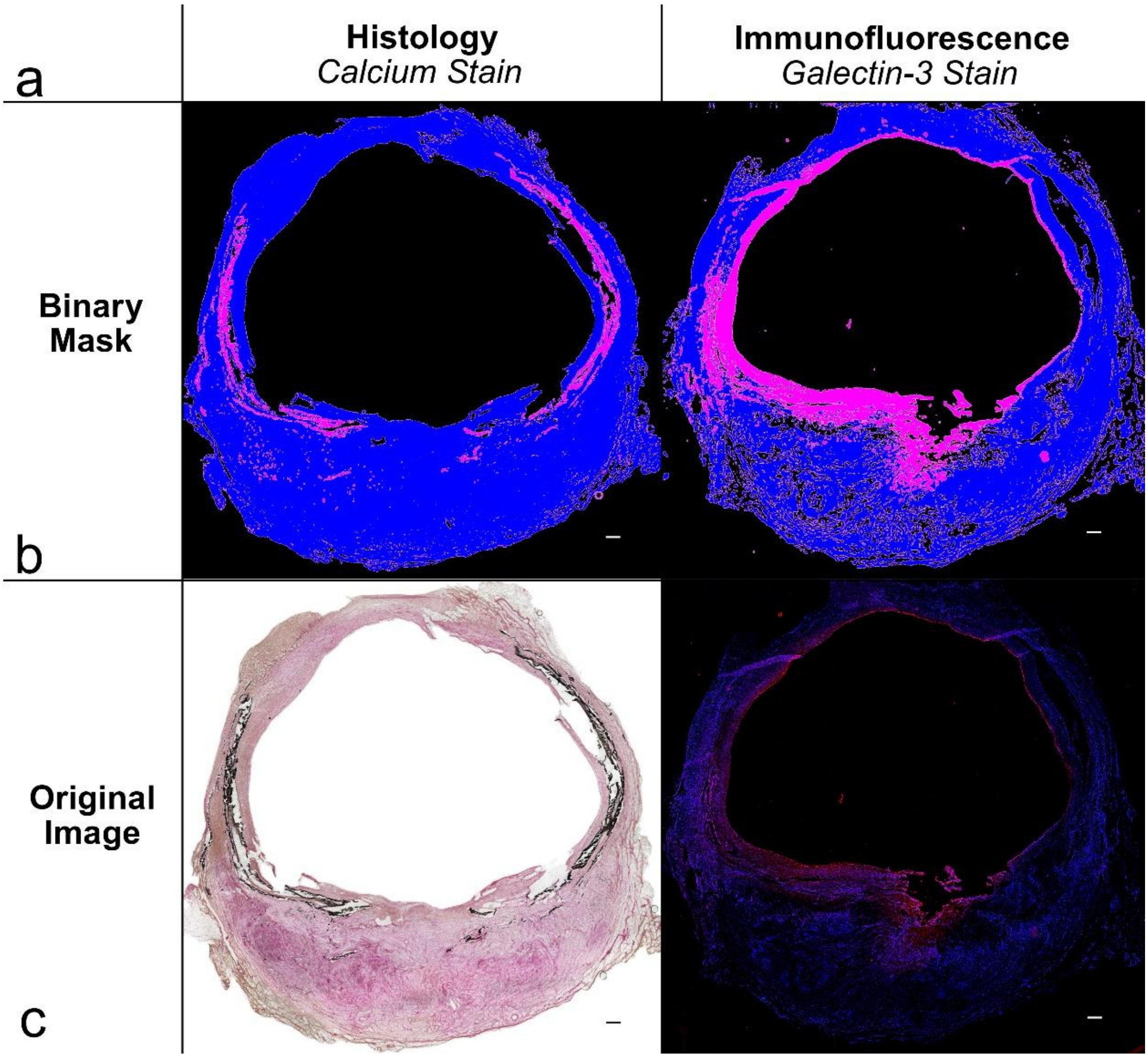


**Fig. S5** Representative examples of automated image segmentation/binary mask output used for quantitative histology and immunofluorescence analysis. (**a**, **b**) Binary masks generated by the Keyence Hybrid Cell Count software (Version 2.2.1; Keyence Corp., Osaka, Japan) illustrate automated pixel classification of positive signal *(pink)* and background tissue *(blue)* for histology *(Von Kossa Calcium stain; left)* and immunofluorescence *(Galectin-3 stain; right)*. (**c**) Corresponding original images before segmentation. Calcium is stained black *(left)*, Galectin-3 antibody-binding is colored pink *(right)* by a fluorescent secondary antibody. Automated thresholding parameters hue, brightness, and color tolerance were kept at the software's default "auto" settings to ensure objective and consistent quantification across different stainings and animals. Scale bars: 500 μm.

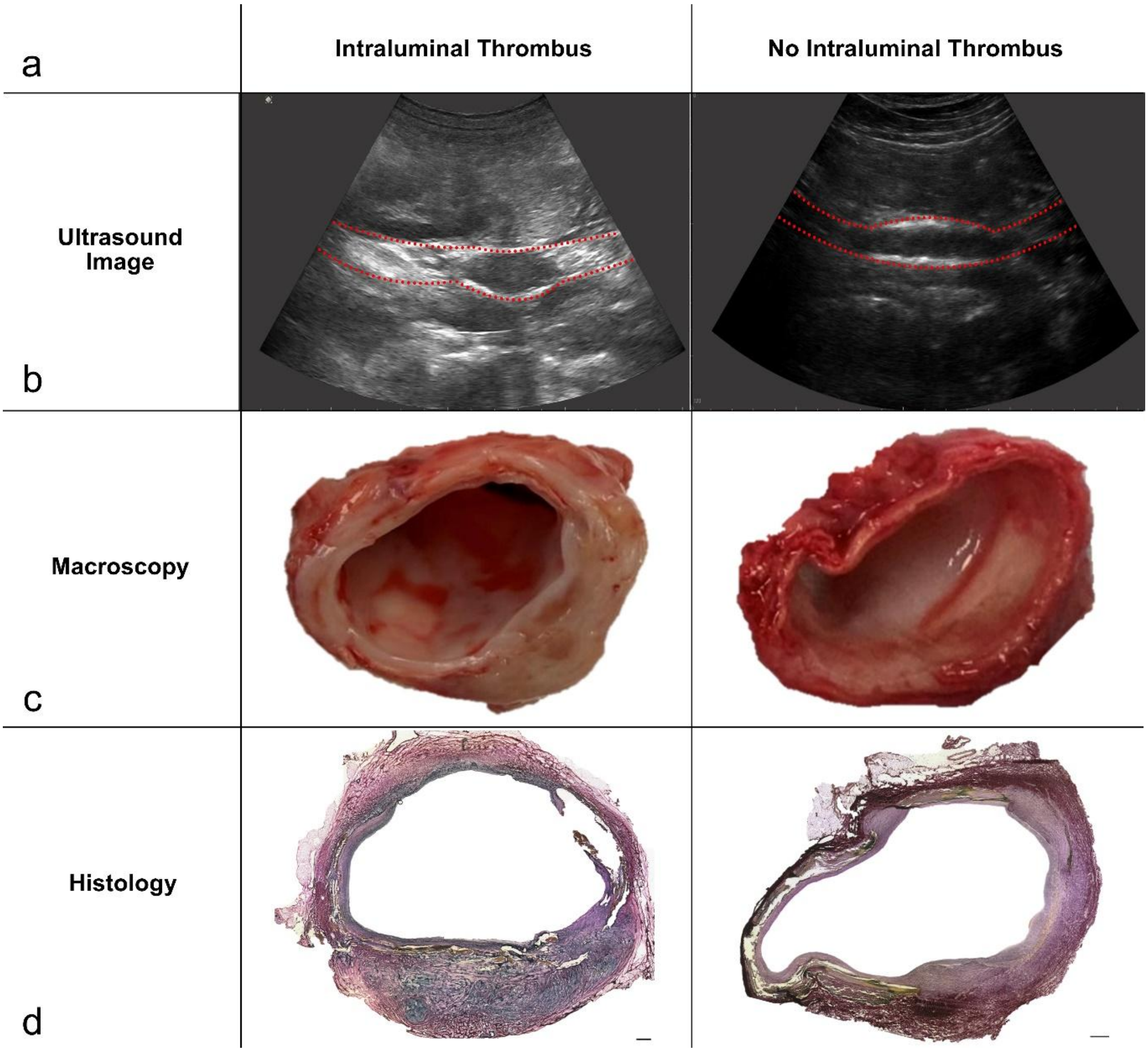

**Fig. S6** Representative examples of aneurysms with and without intraluminal thrombus. (**a**, **b**) Ultrasound images *(outer-to-outer diameter delineated by red dotted lines)* showing an aneurysm containing an intraluminal thrombus (ILT; *left, *thrombus)* and one without ILT *(right).* The echogenic luminal material in the left panel corresponds to thrombus, whereas the right lumen remains echo-free although calcification can be delineated. (**c**) Macroscopic *ex vivo* specimens of the same aortas illustrating the presence *(left, *thrombus)* and absence *(right)* of ILT. (**d**) Histology using Elastica-Van Gieson staining demonstrates layered ILT *(left, *thrombus)* and intact, thrombus-free lumen *(right)*. Scale bars: 500 µm.

**Supplementary figure material**

**See Fig. 6:** Whole blot images for Galectin-3 and α-smooth-muscle-actin

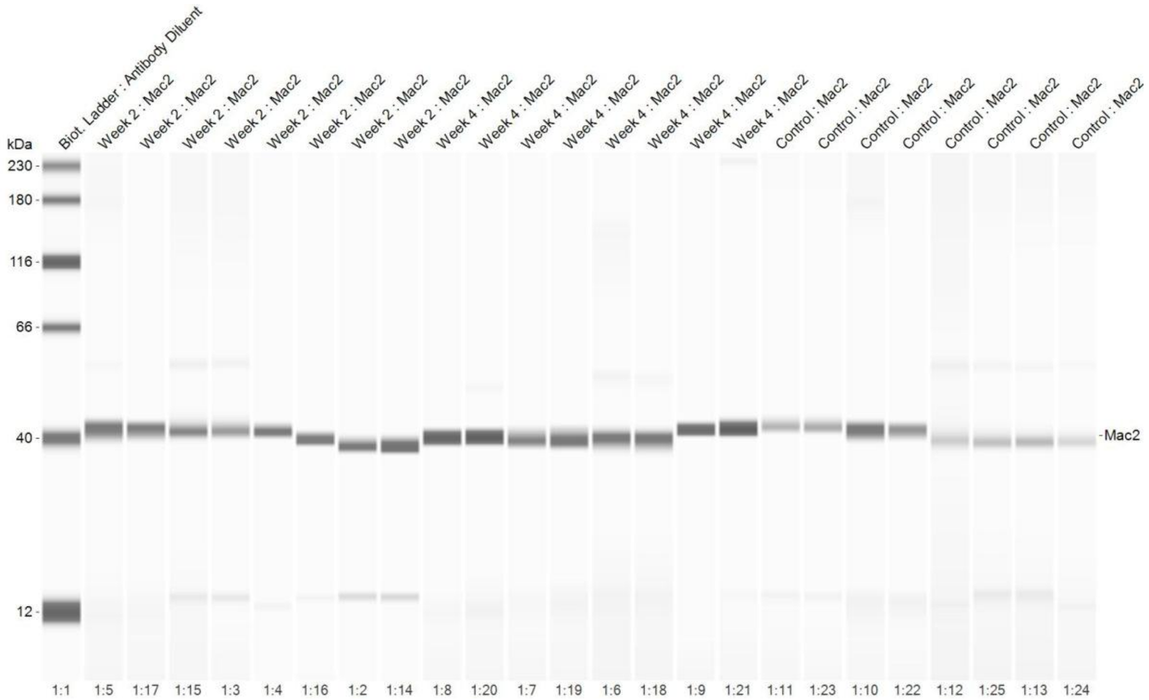


**See Fig. 6a:** Western blot analysis of inflammatory and structural protein expression. Whole blot image for Galectin-3 *(here: Mac2)*, showing two different samples from each animal *(n = 8; group 1: week 2, group 2: week 4)* included in the study. Samples from each animal's non-aneurysmal suprarenal aorta are included as controls. Mac2/Gal-3, Galectin-3.

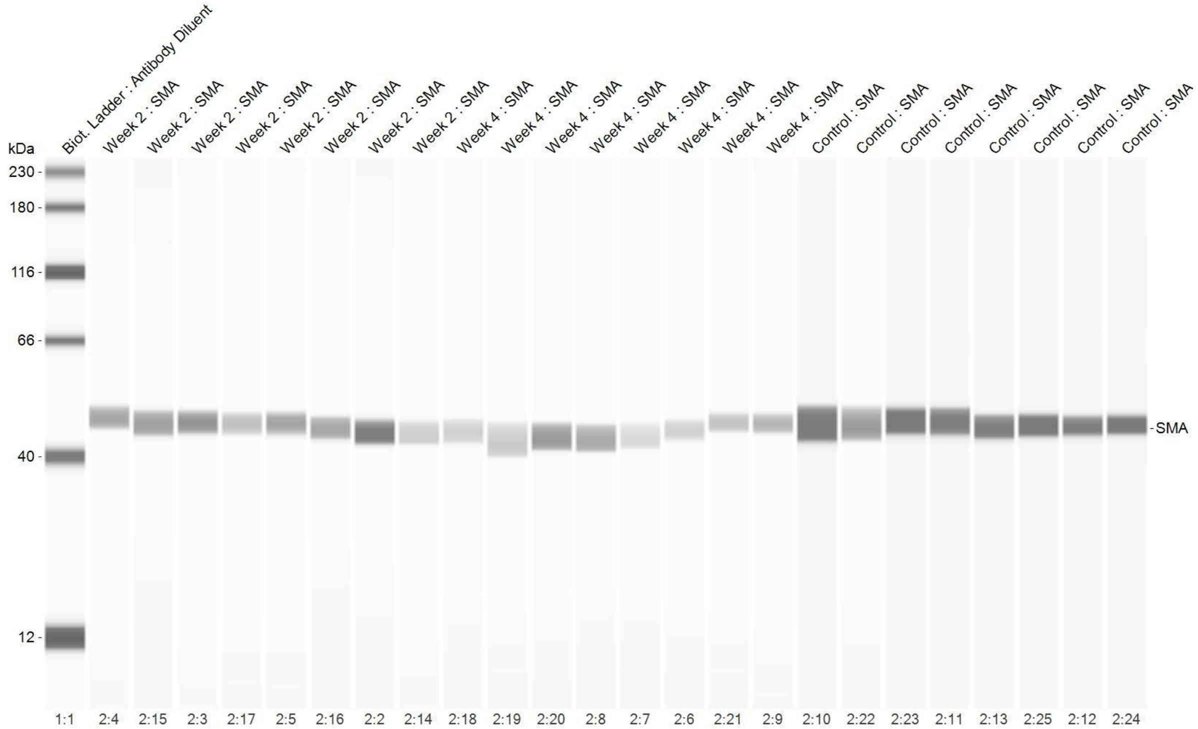


**See Fig. 6c** Western blot analysis of inflammatory and structural protein expression. Whole blot image for α-smooth-muscle-actin *(here: SMA)*, showing two different samples from each animal *(n = 8; group 1: week 2, group 2: week 4)* included in the study. Samples from each animal's non-aneurysmal suprarenal aorta are included as controls. α SMA/SMA, α-smooth-muscle-actin.

**Supplementary images**

*Infrarenal abdominal aorta at necropsy*

**Euthanasia at two weeks post-intervention**

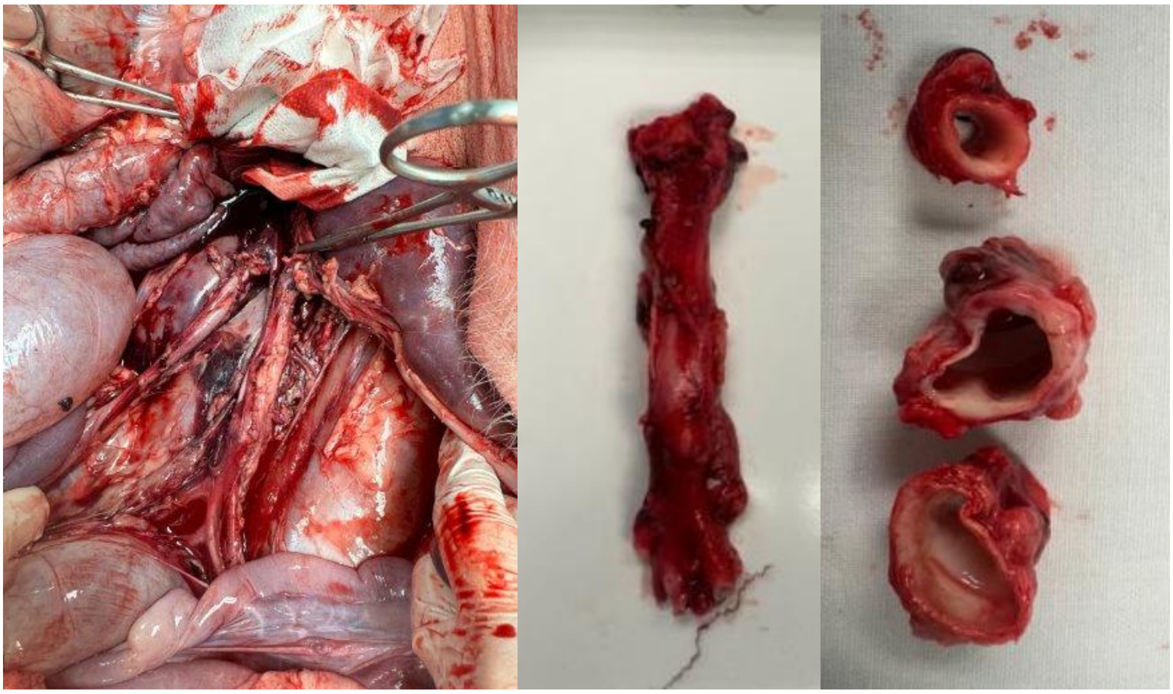

**Animal 1**

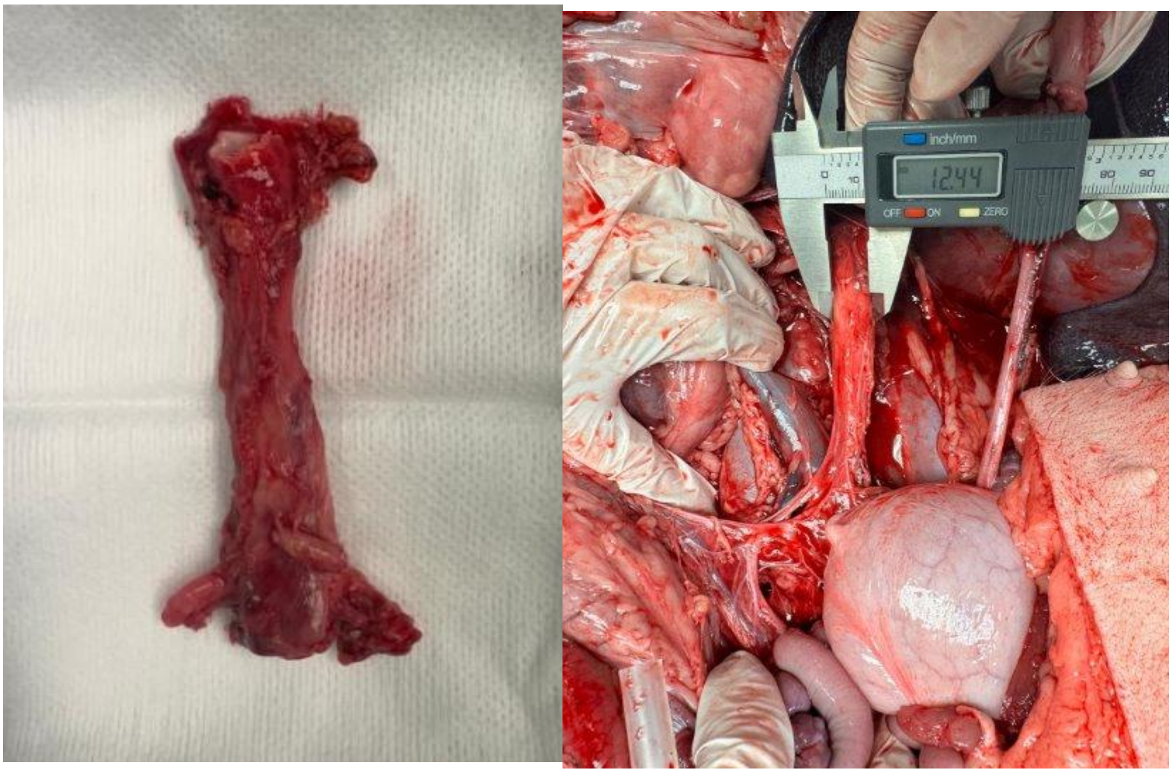


**Animal 2**

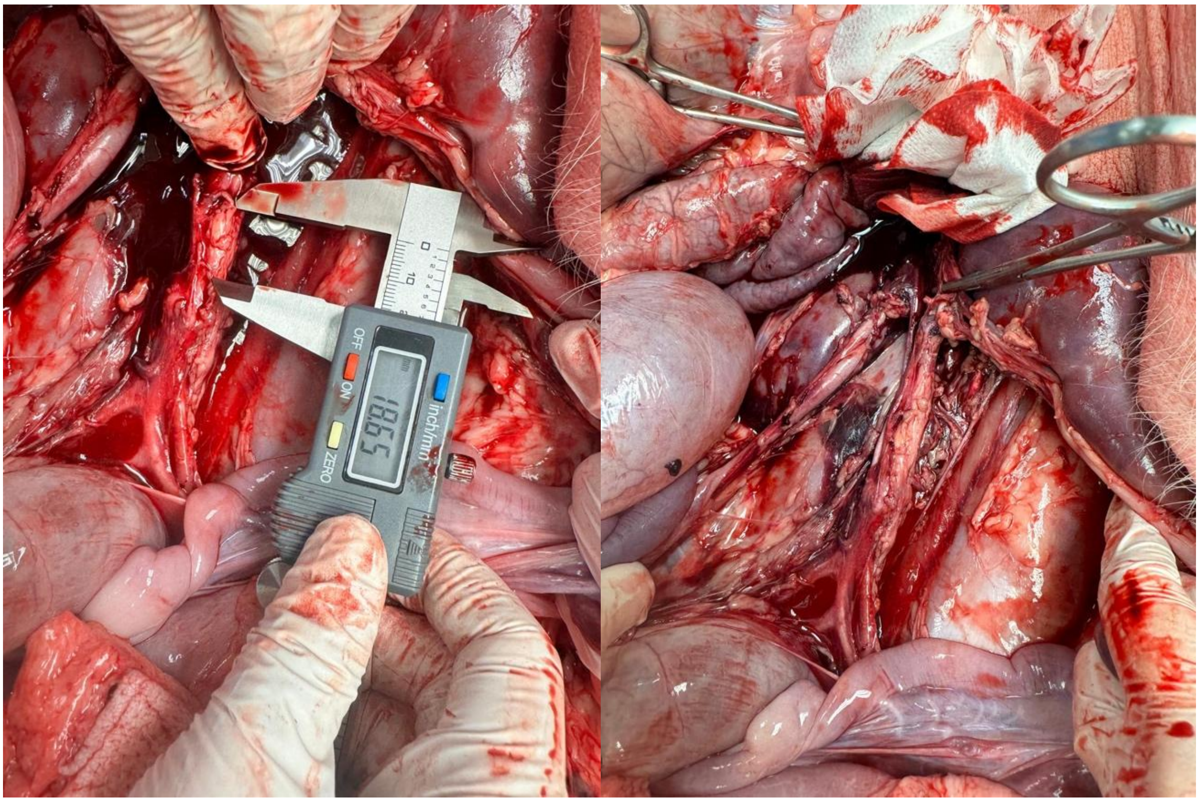


**Animal 3**

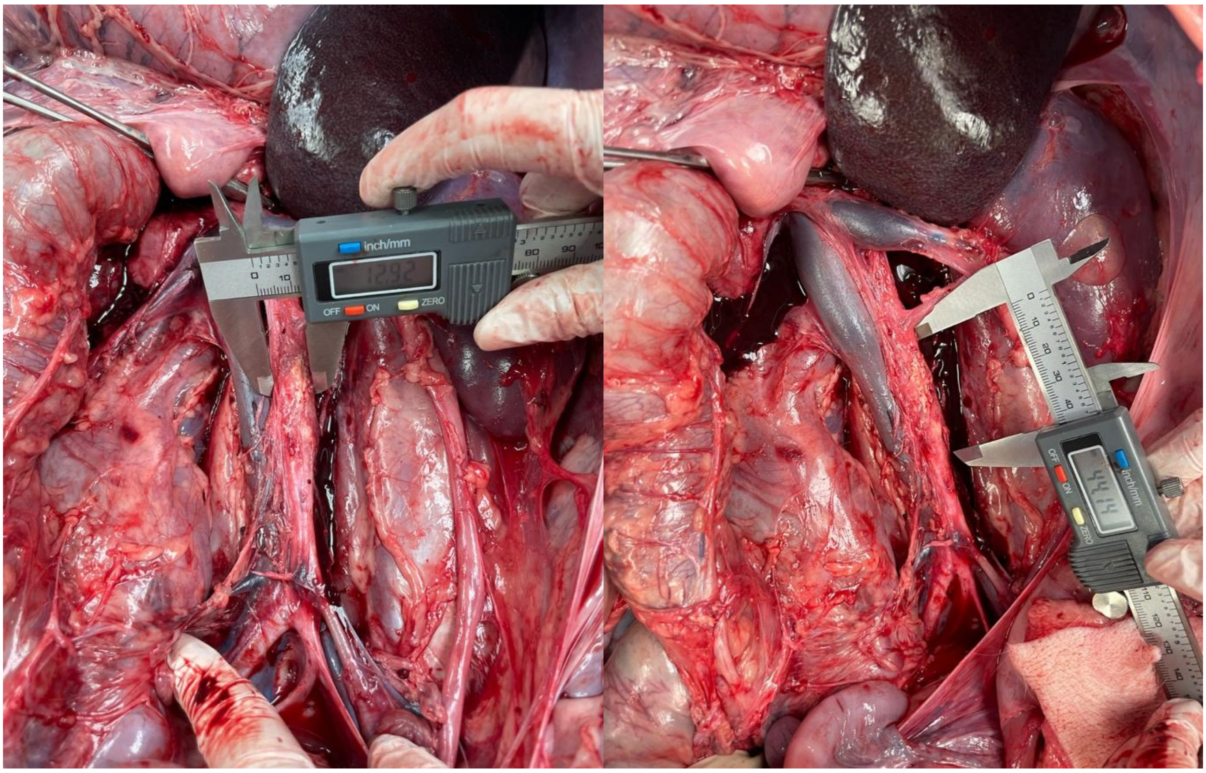


**Animal 4**

**Euthanasia at four weeks post-intervention**

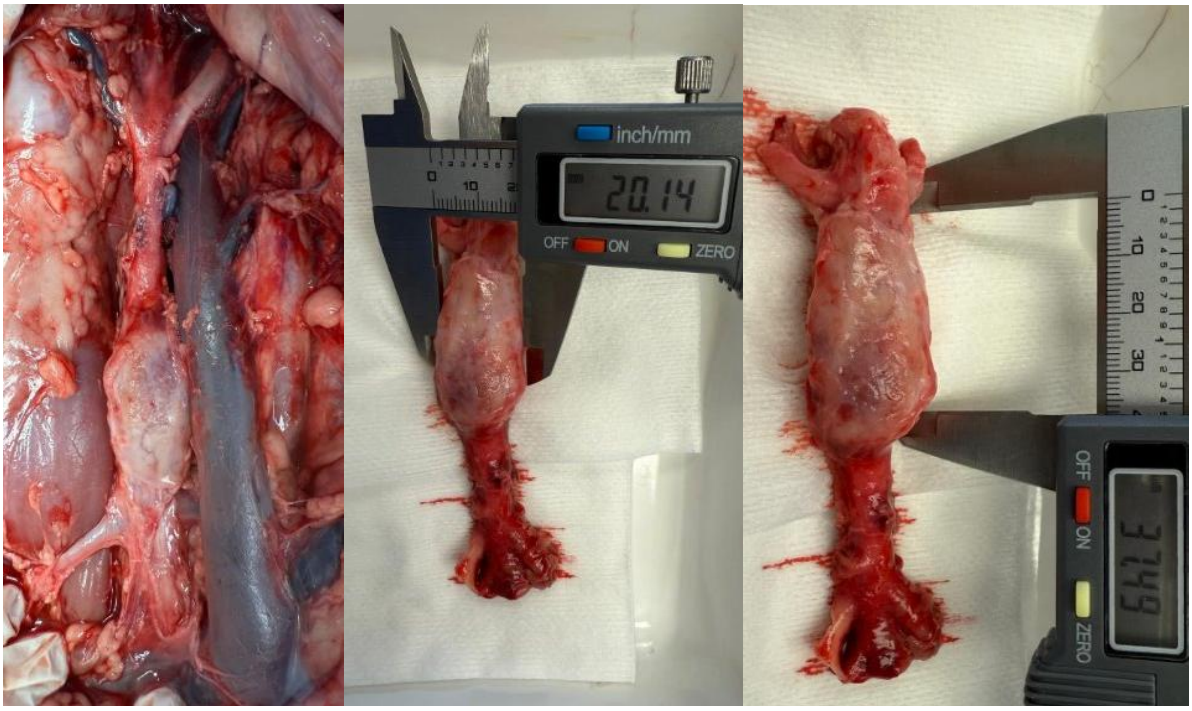


**Animal 5**

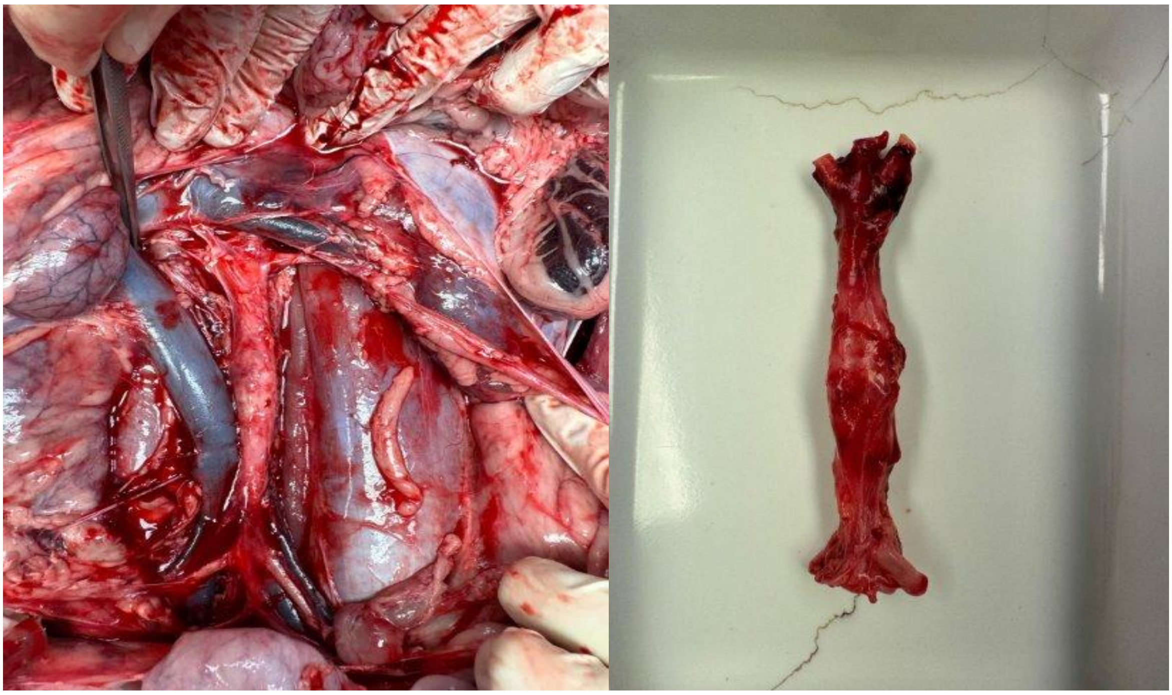

**Animal 6**

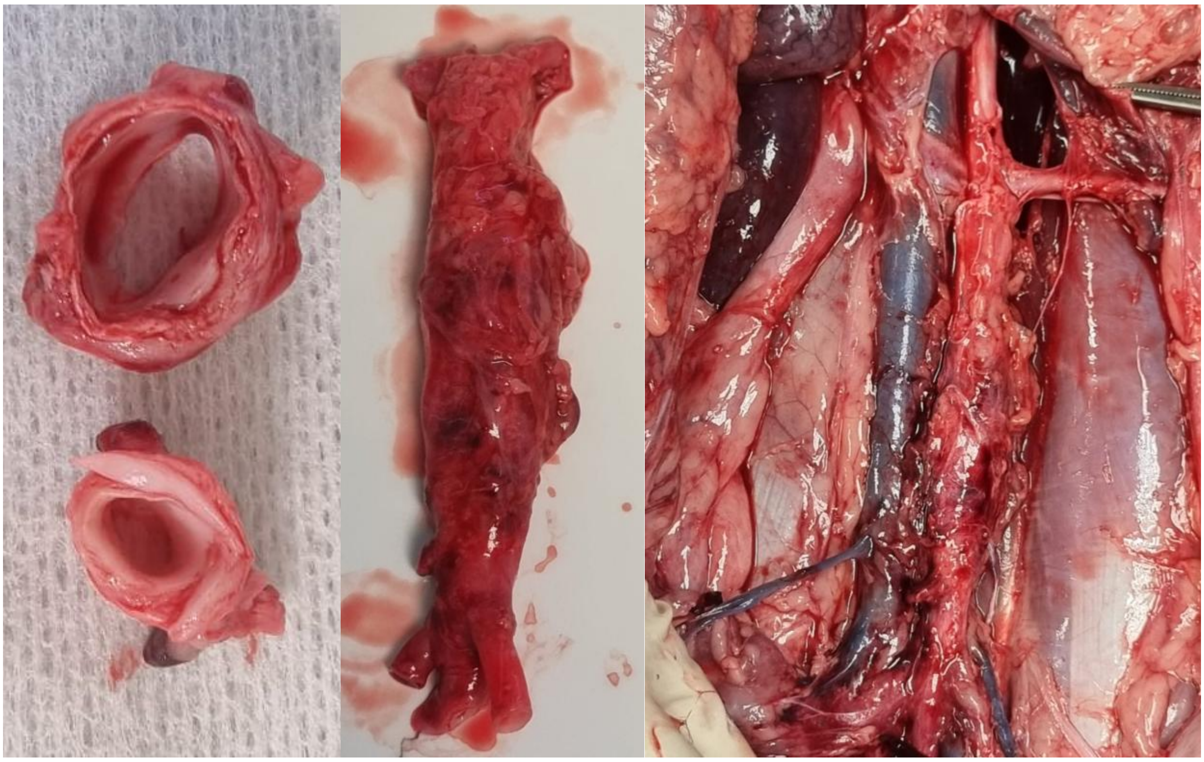

**Animal 7**

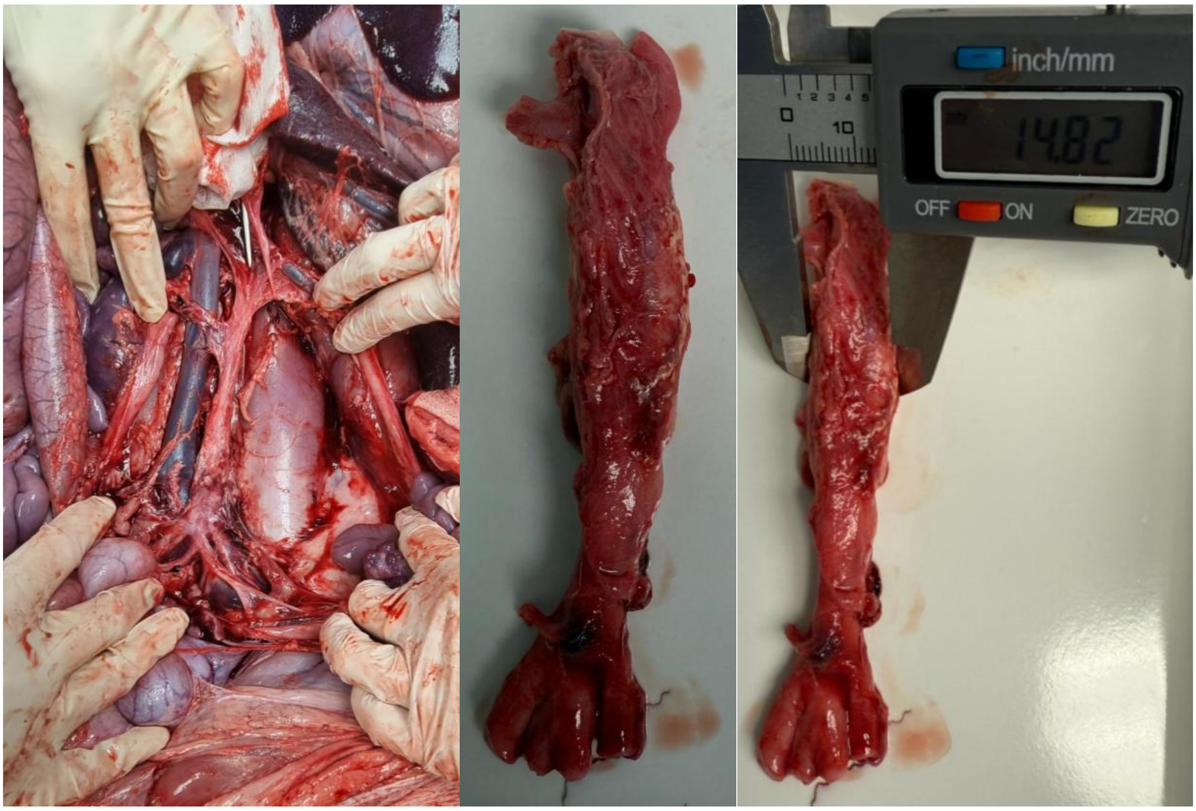


**Animal 8**

**Arterial access sites for interventional procedure**

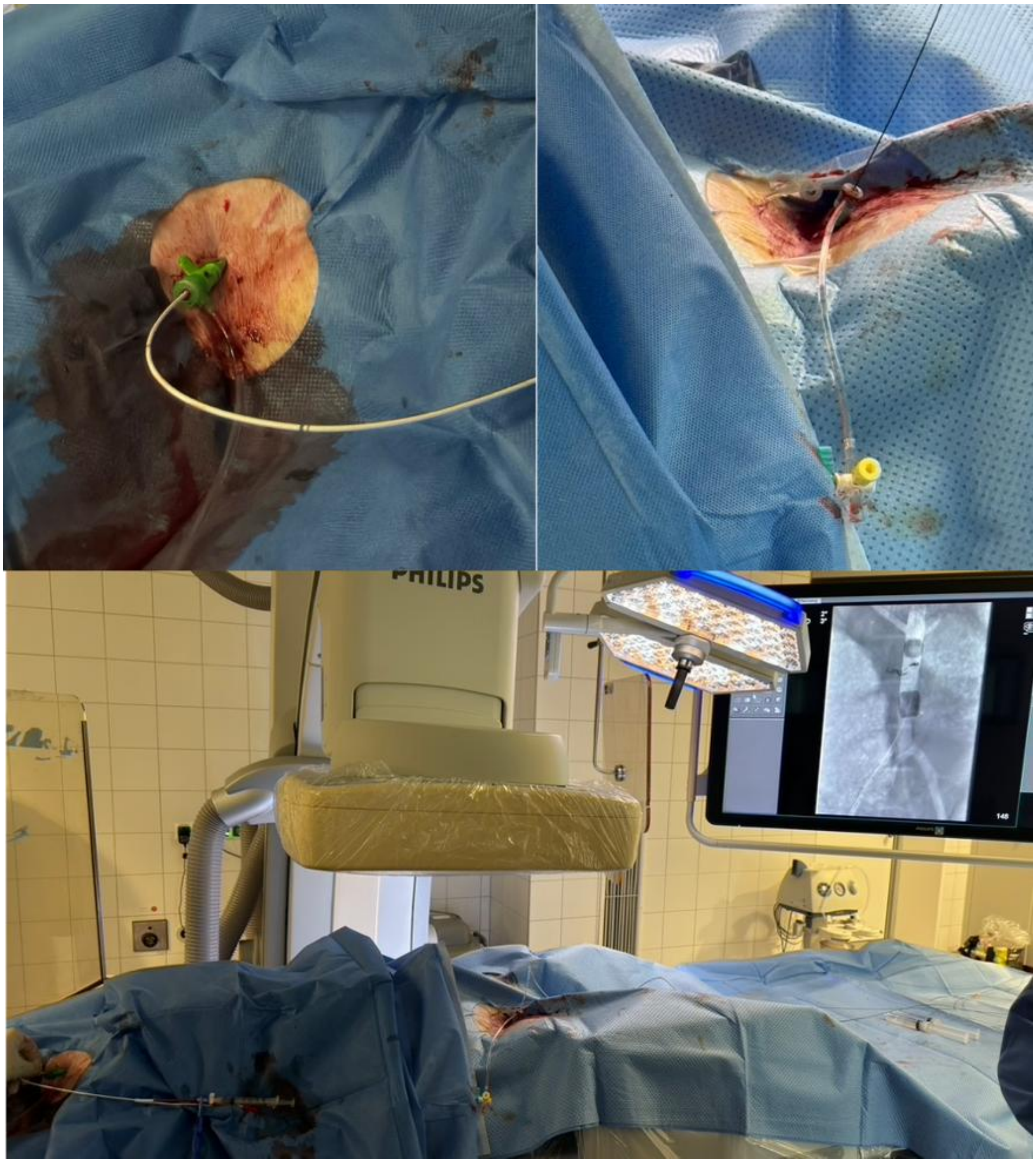


**Endovascular access and angiographic setup for abdominal aortic aneurysm induction.** *(Top left)* Carotid arterial access site with introducer sheath in place following surgical cutdown and catheter insertion. *(Top right)* Femoral arterial access site prepared with vascular sheath and secured under sterile draping. *(Bottom)* Fluoroscopic and angiographic setup showing simultaneous carotid and femoral access during the endovascular procedure, with the angiography system and visualization monitor in the background.

## Supplementary information on materials and medications

**Atropine (Atropinsulfate 0.5 mg/mL):**

#00648037; B. Braun Melsungen AG, Melsungen, Germany.

**Azaperone (Stresnil 40 mg/mL Solution)**:

#BE-V000673; Elanco GmbH, Cuxhaven, Germany.

**Ketamine (100 mg/ml)**:

#1200; CP-Pharma HGmbH, Burgdorf, Germany.

**Xylazine (Xylavet® 100 mg/mL)**:

#401510.01; CP-Pharma HGmbH, Burgdorf, Germany.

**Propofol (1% 10 mg/1 ml MCT)**:

#16661502; Fresenius Kabi Deutschland GmbH, Bad Homburg v. d. Höhe, Germany.

**Propofol (2% 20 mg/1 ml MCT):**

#00194346; Fresenius Kabi Deutschland GmbH, Bad Homburg v. d. Höhe, Germany.

**Fentanyl i.v. Solution (Fentanyl-Hameln 50 µg/mL):**

#06143410; Hameln Pharma GmbH, Hameln, Germany.

**Isoflurane (Isoflurane CP 1 mg/mL):**

#1214; CP-Pharma HGmbH, Burgdorf, Germany.

**Sterofundin® ISO:**

#2506375; B. Braun Melsungen AG, Melsungen, Germany.

**Noradrenaline (Noradrenaline Kabi 1 mg/mL):**

#7000317.00.00; Fresenius Kabi Deutschland GmbH, Bad Homburg v. d. Höhe, Germany.

**Glucose (Glucose B. Braun Vet Care 5 g/100 mL):**

#V7006055.00.0; B. Braun Melsungen AG, Melsungen, Germany.

**Potassium Chloride (Kalium Chlorid 7.45 % MPC Conc. Inj.):**

#03140598; B. Braun Melsungen AG, Melsungen, Germany.

**Fentanyl Patches (Fentanyl-1A Pharma 50 µg/h Matrixpfl.**):

#00682809; 1A Pharma, Novartis AG, Basel, Switzerland.

**Midazolam (Midazolam-ratiopharm® 15 mg/3 mL Inj.):**

#44856.01.00; Merckle GmbH, Blaubeuren, Germany.

**Buprenorphine (TEMGESIC® 0.3 mg/mL Ampules):**

#997.00.00; EUMEDICA Pharmaceuticals GmbH, Lörrach, Germany.

**Heparin (Heparin-Natrium-5000-ratiopharm®):**

#43001343; Merckle GmbH, Blaubeuren, Germany.

**Tromethamine (THAM-Köhler 3 mmol/mL):**

#4699.99.99; Dr. Franz Köhler Chemie GmbH, Bensheim, Germany.

**Metronidazole (Metronidazol Fresenius 500mg/100ml I.v. Solution):**

#05105488; Fresenius Kabi Deutschland GmbH, Bad Homburg v. d. Höhe, Germany.

**Metamizole Ampules (Novaminsulfon-ratiopharm® 500 mg/mL Ampules)**:

#08713863; Merckle GmbH, Blaubeuren, Germany.

**Metamizole Powder (METAPYRIN® Oral 100%):**

#11182642; Serumwerk Bernburg AG, Bernburg, Germany.

**Sulbactam/Ampicillin (Unacid® 2000 mg/1000 mg)**:

#023383-67987-100; Pfizer Pharma GmbH, Berlin, Germany.

**Aqua Ad Injectabilia (Aqua Inject. Miniplasco® Connect Ampules):**

#3113087; B. Braun Melsungen AG, Melsungen, Germany.

**Tris HCl Buffer (1 M Tris-HCl UltraPure™, pH 8.0):**

#15568025; Invitrogen, Thermo Fisher Scientific Inc., Waltham, MA, USA.

**Collagenase (CLS-1 Collagenase, Type 1):**

#LS004194; Worthington Biomedical Corp., Lakewood, NJ, USA.

**Elastase (Elastase 10 mg ≥200 U/mg, lyophilized):**

#39445-21-1; Carl Roth GmbH + Co. KG, Karlsruhe, Germany.

**Calcium Chloride:**

#10043-52-4; Merck KGaA, Darmstadt, Germany.

**PROGREAT® Microcatheter:**

#MC-PE28131ZB; Terumo Medical Corp., Somerset, NJ, USA.

**Foley-Type Urinary Catheter 12F:**

#2310014212; *P*.J. Dahlhausen & Co. GmbH, Cologne, Germany.

**Percutaneous Entry Thinwall Needle:**

18G #BSDN-18-9.0/ 20G #SDN-20-4.0; Cook Group Inc., Bloomington, IN, USA.

**Radifocus™ Vessel Dilator:**

6F RF-VD60K10M/ 8F RF-VD80K10M; Terumo Medical Corp., Somerset, NJ, USA.

**Radifocus™ Introducer II Standard Kit A:**

6F #RS-A60K10AQ/ 8F #RS-A80K10SQ; Terumo Medical Corp., Somerset, NJ, USA.

**TEMPO™ Pigtail Catheter 4F**:

#451-403 L5; Cordis Corp., Hialeah, FL, USA.

**Atlas™ PTA Balloon Catheter (14 mm × 40 mm, 75 cm, 7F)**:

#AT75144; Becton Dickinson GmbH, Heidelberg, Germany.

**Fogarty® Arterial Embolectomy Catheters:**

5.5F #12-080-5FP/ 6F #12-080-6F; Edwards Lifesciences Corp., Irvine, CA, USA.

**ANGIO-SEAL® VIP Vascular Closure Device:**

6F #610130/ 8F #610131; Terumo Medical Corp., Somerset, NJ, USA.

**Ultrasound Gel:**

#4251765100672; medimex GmbH, Limburg a. d. Lahn, Germany.

**Tissue-Tek O.C.T. Compound:**

#4583; Sakura Finetek USA, Torrance, CA, USA.

**MorFFFix® Formaldehyde-Substitute:**

#13616; MORPHISTO GmbH, Offenbach am Main, Germany.

**Verhoeff-Van-Gieson Trichrome Staining Kit:**

#18553; MORPHISTO GmbH, Offenbach am Main, Germany.

**Picro-Sirius Red Staining Kit:**

#13425; MORPHISTO GmbH, Offenbach am Main, Germany.

**Von Kossa Staining Kit:**

#ab150687; Abcam Ltd., Cambridge, UK.

**Acetone ≥ 99 %:**

#L10407.0F; Thermo Fisher Scientific Inc., Waltham, MA, USA.

**Phosphate Buffered Saline, pH 7.4:**

#P4417; Sigma-Aldrich®, Merck KGaA, Darmstadt, Germany.

**Antibody Diluent, Background Reducing):**

#S3022; Agilent Technologies, Santa Clara, CA, USA.

**DAPI Staining Solution (ROTI® Mount FluorCare DAPI):**

#HP19.1; Carl Roth GmbH + Co. KG, Karlsruhe, Germany.

**Goat Anti-Mouse IgG (H+L) Highly Cross-Adsorbed Secondary Antibody, Alexa Fluor™ 647:**

#A-21236; Invitrogen, Thermo Fisher Scientific Inc., Waltham, MA, USA.

**Donkey Anti-Rabbit IgG (H+L) Highly Cross-Adsorbed Secondary Antibody, Alexa Fluor™ 647:**

#A-31573; Invitrogen, Thermo Fisher Scientific Inc., Waltham, MA, USA.

**Galectin-3/Mac2 Antibody:**

#14979-1-AP, 1:10; Proteintech Group Inc., Rosemont, IL, USA.

α-smooth-muscle-actin **Antibody:**

#sc-53142, 1:1000; Santa Cruz Biotechnology Inc., Dallas, TX, USA.

**10% Sodium Dodecyl Sulphate:**

#2326.1; Carl Roth GmbH + Co. KG, Karlsruhe, Germany.

**1 M Tris-HCl:**

#9090.2; Carl Roth GmbH + Co. KG, Karlsruhe, Germany.

**7 M Urea:**

#2317.1; Carl Roth GmbH + Co. KG, Karlsruhe, Germany.

**Glycerol:**

#G5516; Sigma-Aldrich®, Merck KGaA, Darmstadt, Germany.

**Halt™ Protease and Phosphatase Inhibitor Cocktail (100X):**

#78440; Thermo Fisher Scientific Inc., Waltham, MA, USA.

**Pierce™ BCA Protein Assay Kit:**

#23227; Thermo Fisher Scientific Inc., Waltham, MA, USA.

**12–230 kDa Jess™ Separation Module:**

#SM-W001; ProteinSimple™ Biotechne Corp., Minneapolis, MN, USA.

**Jess™ Anti-Rabbit Detection Module:**

#DM-001; ProteinSimple™ Biotechne Corp., Minneapolis, MN, USA.

**Jess™ Anti-Mouse Detection Module:**

#DM-002; ProteinSimple™ Biotechne Corp., Minneapolis, MN, USA.

**Jess™ RePlex™ Module:**

#RP-001; ProteinSimple™ Biotechne Corp., Minneapolis, MN, USA.

**Jess™Total Protein Detection Module:**

#DM-TP01; ProteinSimple™ Biotechne Corp., Minneapolis, MN, USA.
